# The Escalating Threat of Heatwaves in Central Asia: Climate Change Impacts and Public Health Risks

**DOI:** 10.1002/gch2.202500401

**Published:** 2025-11-08

**Authors:** Parya Broomandi, Mehdi Bagheri, Ali Mozhdehi Fard, Mostafa Hadei, Mohammad Abdoli, Adib Roshani, Aram Fathian, Sadjad Shafiei, Michael Leuchner, Prashant Kumar, Jong Ryeol Kim

**Affiliations:** ^1^ Department of Civil and Environmental Engineering School of Engineering and Digital Sciences Nazarbayev University Kabanbay Batyr Ave. 53 Astana 010000 Kazakhstan; ^2^ Department of Electrical and Computer Engineering School of Engineering and Digital Sciences Nazarbayev University Kabanbay Batyr Ave. 53 Astana 010000 Kazakhstan; ^3^ Department of Environmental Health Engineering School of Public Health Tehran University of Medical Sciences Tehran 417613151 Iran; ^4^ Climate Change and Health Research Center (CCHRC) Institute for Environmental Research (IER) Tehran University of Medical Sciences Tehran 1439813118 Iran; ^5^ Physical Geography and Climatology Department of Geography RWTH Aachen University Wüllnerstr. 5b 52056 Aachen Germany; ^6^ Faculty of Civil Engineering Babol Noshirvani University of Technology Babol 484 Iran; ^7^ Water, Sediment, Hazards and Earth‐surface Dynamics (waterSHED) Lab Department of Geoscience University of Calgary Calgary Alberta T2N 1N4 Canada; ^8^ Global Centre for Clean Air Research (GCARE) School of Sustainability, Civil and Environmental Engineering Faculty of Engineering and Physical Sciences University of Surrey Guildford Surrey GU2 7XH UK; ^9^ Institute for Sustainability University of Surrey Guildford Surrey GU2 7XH UK

**Keywords:** all‐cause mortalities, Mann–Kendall trend analysis, middle of the road scenario, sustainable urban development goals, urban overheating

## Abstract

Extreme temperature events, particularly heatwaves, are intensifying due to climate change and urbanization, posing major public health challenges in Central Asia (CA), where research is limited. Despite the rising frequency and severity of heat extremes, long‐term assessments of their health impacts are scarce. This study addresses this gap by analyzing historical and future heatwave trends and associated health risks using multi‐ensemble climate models across 700 locations from 1959 to 2100. Bias correction improved GCMs, reducing bias and RMSE by 24% and 14%, respectively. Under SSP2–4.5, projected heatwave magnitudes (HWM) shift from 26 to 31 °C, consistent with historical moderate to severe events. Under SSP5–8.5, HWM increases to 29–36 °C. Turkmenistan is expected to experience ultra‐extreme heatwaves in the far future, a pattern not seen in other CA countries. Under SSP2–4.5, Kazakhstan and Uzbekistan show the highest rises in heatwave‐related mortality rates, with slopes of 5.432 and 3.021 in the near future, declining to 1.377 and 1.102 in the far future. SSP5–8.5 shows similar but higher estimates, highlighting escalating public health risks. Findings emphasize the urgent need for region‐specific climate policies and public health strategies to mitigate the growing burden of extreme heat in CA.

## Graphical abstract

Extreme heatwaves in Central Asia are intensifying due to climate change and urbanization, amplifying public health risks. Using bias‐corrected climate model ensembles across 700 locations (1959–2100), this study projects substantial increases in heatwave magnitude, with Turkmenistan facing ultra‐extreme events under SSP5–8.5. Kazakhstan and Uzbekistan are identified as hotspots for rising heatwave‐related mortality. These findings highlight the escalating health burden of extreme heat in Central Asia and underscore the urgent need for targeted climate policies and public health strategies.

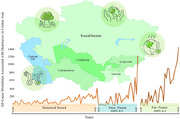

## 1 Introduction

Millions of individuals across the globe are impacted by natural disasters.^[^ ^1^, ^2^ ^]^ Nonetheless, the effects of extreme weather events are not consistent across different regions, and understanding their localized characteristics is essential for effective risk mitigation. Climate change is altering the frequency and intensity of weather extremes such as heatwaves, floods, and droughts, which is driven by influential factors altering the global energy balance, such as anthropogenic greenhouse gas emissions and land use changes.^[^ ^3^, ^4^, ^5^ ^]^

Weather extremes, focusing on heatwaves, can have significant negative impacts on human life in society.^[^ ^6^, ^7^ ^]^ A notable rise in the frequency of heatwaves has been reported worldwide,^[^ ^8^ ^]^ for example, in western Russia, western Europe, and eastern China during summertime. Consequently, a mounting number of widespread droughts, heat‐related death numbers, and extensive crop losses have been observed worldwide.^[^ ^9^, ^10^ ^]^ This situation underscores the pressing necessity for enhanced forecasting and adaptation strategies.^[^ ^9^ ^]^

Heatwaves are particularly critical in regions with arid and continental climates, where limited soil moisture and high radiative forcing can amplify temperature extremes.^[^ ^5^, ^11^ ^]^ CA represents one of the world's most climate‐sensitive dryland regions, combining rapidly rising background warming with water scarcity, strong land–atmosphere feedbacks, and fragile socioeconomic systems.^[^ ^12^, ^13^ ^]^ These characteristics make the region highly prone to both physical intensification of heatwaves and cascading health and economic impacts.

In CA, multiple studies have observed an increasing trend in the temperature with a more rapid rate compared to global averages, escalating in its intensity noted in the mid‐1990s.^[^ ^14^, ^15^, ^16^ ^]^ Across CA, a high vulnerability to the adverse impacts of climate change has been reported.^[^ ^3^, ^12^ ^]^ A higher rise of temperature (≈0.30 °C per decade), compared to the global average (0.19 °C per decade), was observed over the past fifty years in CA.^[^ ^17^ ^]^

Upcoming events that are exceptionally long, intense, and frequent will create circumstances that will impact the well‐being of humans and civilizations, and have significant effects on livestock, agriculture, and wildlife. The region is experiencing significant warming and a shift toward hotter conditions, mostly caused by the increased greenhouse effect resulting from ongoing human activities such as the release of greenhouse gases and changes in land use.^[^ ^9^, ^16^, ^18^, ^19^ ^]^

Despite these alarming trends, research related to both historical and anticipated climate change impacts in CA remains markedly insufficient, thereby leading to a considerable knowledge gap.^[^ ^18^, ^20^, ^21^, ^22^ ^]^ The majority of research is centered on examining alterations in mean temperature and precipitation in CA. While temperature changes exhibit a uniform and distinct pattern across the region, precipitation variations display significant heterogeneity. In addition, a limited number of studies by investigating the temperature and precipitation distributions have highlighted the urgent need for focusing on extreme weather events such as heatwaves.

The lack of region‐specific research on heatwaves, their expected trends, and associated dangers poses a considerable challenge to climate adaptation initiatives in this region. Previous research, however limited, suggests that heatwaves in CA are expected to intensify, increase in frequency, and extend in duration. This trend is expected to exacerbate hazards to public health and socioeconomic stability.^[^ ^23^ ^]^ Without a comprehensive understanding of the changing dynamics of heatwaves, the region is highly vulnerable to unexpected climatic and socioeconomic consequences.

Recent evidence highlights the vital importance of this issue. In June 2021, Kazakhstan recorded exceptional temperatures, reaching 46.5 °C in the Kyzylorda, Mangystau, and Turkistan regions. This extraordinary temperature caused extreme drought conditions, leading to the loss of almost 2000 animals owing to shortages in both forage and water.^[^ ^24^ ^]^ Furthermore, a survey released by UNICEF in 2024 revealed that over 92 million children in CA and Eastern Europe are acutely exposed to prolonged heatwaves, putting this region twice as susceptible as the global average.^[^ ^25^ ^]^ Moreover, climate models predict that average temperatures in CA could rise by up to 6.5 °C by the century's end. This acceleration is expected to lead to irreversible climatic alterations, significant aridity, and a considerable increase in occurrences of severe heat waves.^[^ ^26^ ^]^

This research aims to provide a comprehensive assessment of the geographical and temporal variations of heatwaves across CA, employing multi‐model ensemble projections from the Coupled Model Intercomparison Project Phase 6 (CMIP6) to address existing knowledge gaps. This research provides an extensive analysis of extreme temperature occurrences and their direct impacts on public health and regional climate resilience, in contrast to previous studies that mostly focus on changes in mean temperature. Given the expected trends of more severe, frequent, and prolonged summer heatwaves, an in‐depth assessment of heatwave patterns, severity, and distribution is crucial for developing effective adaptation strategies.

Despite these vulnerabilities, CA has been underrepresented in global and regional climate studies. Most prior work has emphasized mean temperature and precipitation changes, e.g.,^[^ ^17^, ^22^ ^]^ while relatively few studies have systematically investigated heatwaves in CA. Moreover, explicit assessment of health impacts associated with projected heatwaves remains absent for the region, despite evidence from other parts of the world that heat extremes significantly increase mortality and morbidity.^[^ ^27^ ^]^ This gap underscores the importance of region‐specific analyses linking climate extremes to human health outcomes.

This research utilizes multi‐CMIP6 GCM ensemble predictions to: 1) investigate spatiotemporal changes in heatwaves and identify highly vulnerable regions; 2) analyze the distribution of warm days and nights across CA; and 3) assess the long‐term evolution of heatwave‐associated health risks from 1959 to 2100. This study offers innovative insights into heatwave dynamics, providing essential direction for decision‐makers and policymakers in formulating effective heat mitigation methods and adaptation frameworks specific to CA's unique climatic challenges.

In contrast to existing CMIP6‐based studies that have primarily examined mean temperature and precipitation changes in CA^[^ ^17^, ^22^ ^]^ or provided limited downscaling of heat extremes without explicit health assessments,^[^ ^7^ ^]^ our study provides one of the first region‐wide, high‐resolution evaluations of heatwave characteristics using 700 stations. Moreover, we explicitly link heatwave projections to all‐cause mortality estimates, offering an integrated climate–health perspective that has not been previously addressed for this region. This combination of bias‐corrected projections, multiple heatwave indices, and health impact quantification represents a novel contribution that advances both scientific understanding and practical adaptation planning in CA.

## 2 Experimental Section

### 2.1 Study Domain

**Figure 1:**
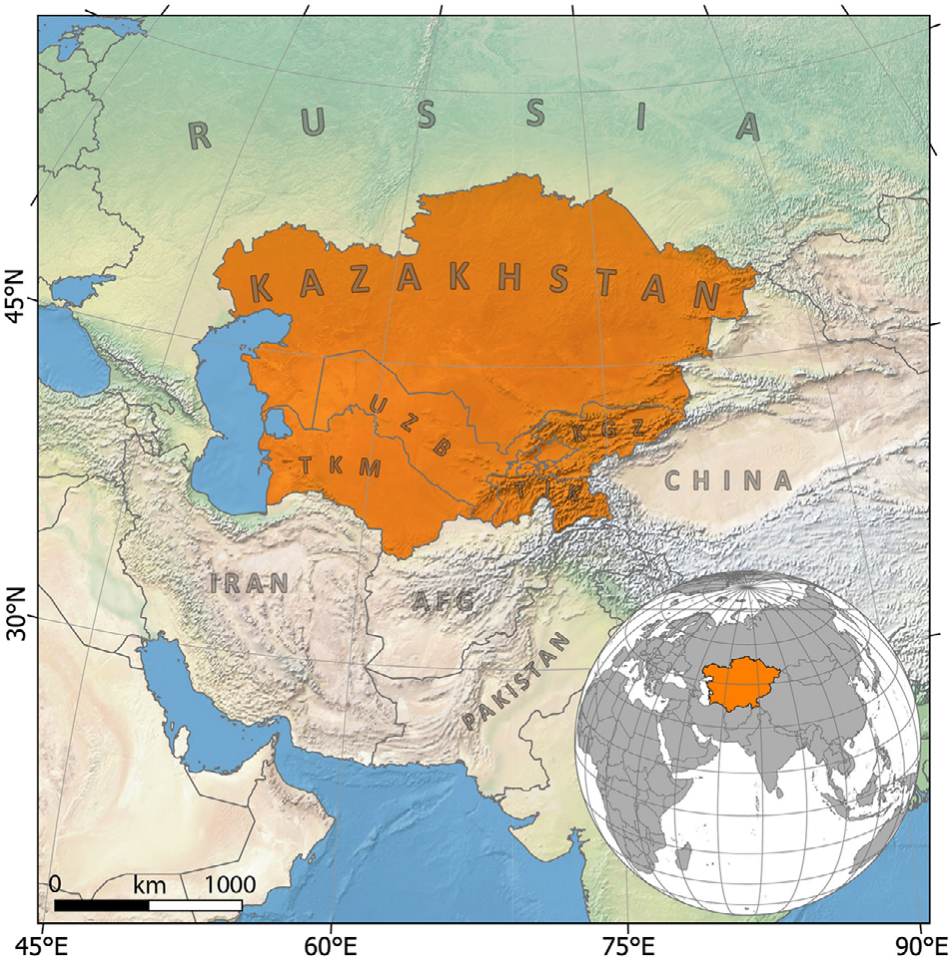
Physiographic map of CA (study area) encompassing Kazakhstan, Kyrgyzstan, Tajikistan, Turkmenistan, and Uzbekistan.

CA consists of the five countries of Uzbekistan, Kazakhstan, Kyrgyzstan, Tajikistan, and Turkmenistan (**Figure** **1**). This area is characterized by arid areas and grasslands, which are shifting from steppes in the northern parts to semi‐desert in the southern regions. Notable annual temperature fluctuations, limited amounts of precipitation, and high evaporation rates are observed in vast parts of CA. Particularly, countries of Uzbekistan, Turkmenistan, and Kazakhstan with lower elevations experience the aforementioned changes. While countries experiencing lower mean temperatures and more precipitation are located in mountains areas such as Tajikistan and Kyrgyzstan.^[^ ^28^ ^]^ The population of CA is ≈66.3 million, with the lowest population density in Kazakhstan and the highest population density in Uzbekistan.^[^ ^23^ ^]^ Additionally, CA has faced a notable increasing trend in warming. Surface air temperatures increased between 1979 and 2011 with an increase of 0.36 to 0.42 °C per decade.^[^ ^29^, ^30^ ^]^ The average annual temperature increased by ≈5.78 °C in Kazakhstan between 1901 and 2016.^[^ ^30^ ^]^ Moreover, the mean temperature has increased from 4.8 °C to 6 °C over the past two decades in Kyrgyzstan.^[^ ^30^, ^31^ ^]^

### 2.2 Data

In the current study, the ERA5 (ECMWF Reanalysis v5) reanalysis database was used to obtain daily basis meteorological data including the average daily air temperature (minimum and maximum) (°C), and daily precipitation sums (mm day^−1^).^[^ ^32^ ^]^ The data were collected from a total of 700 locations in CA covering the geographical areas of urban centers, suburban areas, and rural areas between 1959 and 2021. Based on the data availability and the improved reliability of observational datasets, the 1959–2021 period was selected to ensure a sufficiently long record to capture climate variability and trends.

Furthermore, General Circulation Models (GCMs), CMIP6, with different spatial resolutions, were selected and downloaded from the C3S center to investigate the impact of climate change on variables of interest. In this study, historical outputs of the CMIP6 models were selected during 1959–2021, and simulation outputs are selected during two future periods including near future (2022–2051) and far future (2071–2100) (Table S1, Supporting Information). To evaluate the short‐ to mid‐term climatic risks related to adaptation planning, the near future period (2022–2051) has been designated based on the established climatological standards by the World Meteorological Organization (WMO).^[^ ^33^ ^]^ To encompass the long‐term climate projections, the far future period (2071–2100) was selected. The selected timeframe, by assuring coherence with global climate research, offers valuable insights into continuing warming trends and their associated impacts.^[^ ^12^, ^32^ ^]^

We employed the Inverse Distance Weighting (IDW) algorithm for interpolation, generating raster outputs with a spatial resolution of 0.1° by 0.1°. This approach was selected as it provides a straightforward and reliable way to visualize spatial gradients while preserving the overall structure of the original datasets. It is important to emphasize that all analytical procedures were conducted using the datasets at their original spatial resolutions—ERA5 at 0.25°, and CMIP6 at their respective native resolutions, which vary depending on the model. Moreover, the four nearest points were utilized for the interpolation calculation. To visualize all groups of maps together with a single‐color bar, the upper and lower limits of the color bar were determined by the 2nd and 98th percentiles, respectively, considering all maps. For map groups containing both negative and positive values, the color bar limit was set to the  ± maximum of the absolute values of the 2nd and 98th percentiles, with the center of the color bar set to zero.

### 2.3 Calculation of Climate Indices

The climatic indices proposed by the WMO's Climatology Commission^[^ ^34^ ^]^ were utilized to monitor severe climatic situations. Specifically, heatwave‐related indices were calculated based on the 90th percentile of daily maximum temperature (TX90). These include Heatwave Duration (HWD, number of consecutive days exceeding the threshold), Heatwave Frequency (HWF, number of days per year above the threshold), Heatwave Number (HWN, count of discrete heatwave events per year), Heatwave Magnitude (HWM, an integrated measure of intensity and duration), and Heatwave Amplitude (HWA, the maximum intensity within events). The software tool ClimPACT2 (available at https://climpact‐sci.org) was employed to compute and assess these indices. Full definitions and units are provided in Table S2 (Supporting Information) and are detailed further in the Supporting Information.

The Mann–Kendall nonparametric test was used to assess the statistical significance of the observed trends, with a significance threshold of 5% (*p*‐value < 0.05) applied to determine significant trends.^[^ ^35^, ^36^ ^]^ Moreover, we assessed the severity of heatwaves by classifying their magnitude.^[^ ^37^, ^38^ ^]^ It is possible to quantify the severity of events in a single metric using heatwave magnitude, considering their duration and temperature anomalies.^[^ ^38^ ^]^

**Table** **1** presents a detailed categorization of heatwave episodes according to the heatwave magnitude (HWM) values.^[^ ^38^, ^39^, ^40^ ^]^ HWM are classified according to the following definitions given in Table 1.

**Table 1:** Levels of yearly HWM across CA between 1959 and 2021 (historical period of study). Please note that the classification of HWM into categories (Normal, Severe, Extreme, Super Extreme, Ultra‐Extreme) follows the scheme proposed by Zittis et al.,^[^ ^41^ ^]^ Table 1, ensuring consistency and comparability with previous studies.

HWM [°C]	Heatwave category	HWM [°C]	Heatwave category
0	No heatwave	32–38	Extreme^d)^
12–23	Normal^a)^	38–39	Very extreme^e)^
23–31	Moderate^b)^	≥ 40	Super extreme^f)^
31‐32	Severe^c)^	≥ 50	Ultra‐extreme^g)^

^a)^ Normal: All the grid points show at least one heatwave with 12 °C < HWM ≤ 23 °C of CA during the historical period;

^b)^ Moderate: At least one heatwave with 23 °C < HWM≤ 31 °C is detected in 80% of CA during the historical period;

^c)^ Severe: At least one heatwave with 31 °C< HWM ≤ 32 °C is detected in 70% of CA during the historical period;

^d)^ Extreme: At least one heatwave with 32 °C < HWM ≤ 38 °C is detected in 30% of CA during the historical period;

^e)^ Very extreme: At least one heatwave 38 °C < HWM≤39 °C is detected in 3% of CA during the historical period;

^f)^ Super extreme: HWM ≥ 40 °C;

^g)^ Ultra Extreme: HWM ≥ 50 °C.

### 2.4 Climate Change: Statistical Analysis of GCM Models and Different Scenarios

Future projections of climatic indices in CA utilized multiple GCMs.^[^ ^42^ ^]^ Under the Shared Socioeconomic Pathway (SSP), two climate projections of SSP2–4.5, and SSP5–8.5 scenarios in the CMIP6 archive were used to address uncertainty related to future social conditions. Among the available scenarios, SSP2–4.5 and SSP5–8.5 were chosen because they represent two contrasting climate futures—an intermediate stabilization pathway (SSP2–4.5) and a high‐emission trajectory (SSP5–8.5)—allowing for a robust assessment of both moderate and extreme climate outcomes relevant to compound heatwave hazard in CA.

In this study, we focused on SSP2–4.5 and SSP5–8.5, which are widely used in regional climate impact assessments. Together, they represent a broad range of plausible futures, from intermediate stabilization to high‐emission trajectories. Other scenarios, including SSP1–2.6 and SSP3–7.0 were not included due to computational and data management constraints.^[^ ^12^ ^]^

GCM outputs were subject to bias correction using observed data sources due to modeling discrepancies, and spatial resolutions, and downscaled to align with requirements for climate change impact assessments.

Through Equation (1), the CMIP6 GCM outputs from 1959 to 2100 were analyzed using a hybrid semi‐parametric technique, which was chosen for its simplicity and computational efficiency.^[^ ^43^ ^]^

(1) $${\hat{X}}_{m}^{\prime }=\mu_{o}+\frac{\sigma_{o}}{\sigma m}\left(X_{m}^{\prime }-\mu_{m}\right)$$

${\hat{X}}_{m}^{\prime }$, $X_{m}^{\prime }$, µ_*o*_, µ_*m*_, σ_*o*_, and σ_*m*_.correspond to bias corrected simulated future mean values, future mean values, historical mean values, simulated historical mean values, historical standard deviation, and simulated historical standard deviation, respectively.^[^ ^43^ ^]^

To address the discrepancies among distinct GCMs, the multi‐GCM ensemble averages were computed. The method of “one model, one vote” weighting scheme, through the equal‐weight averaging of bias‐corrected projections from various GCMs, was applied to reduce the inherent uncertainty associated with each individual GCM model.^[^ ^43^ ^]^

### 2.5 Assessment of Bias Correction Performance

To statistically assessment of the performance of model in simulating the interest variables, the bias and root mean square (RMSE) were computed,^[^ ^17^, ^44^, ^45^, ^46^ ^]^ using ERA5 reanalysis data as the reference.

(2) $$Bias=\frac{1}{N}\sum_{i=1}^{n}\left(b_{i}-o_{i}\right)$$

(3) $$RMSE=\sqrt{\sum_{i=1}^{n}\frac{{\left(b_{i}-o_{i}\right)}^{2}}{n}}$$

### 2.6 Health Impact Assessment

The health implications of heatwaves were assessed using an approach adapted from Nori–Sarma et al.^[^ ^47^ ^]^ In contrast to their method, which applied propensity score matching with daily mortality records, we employed pooled relative risk (RR) values from published meta‐analyses due to the absence of CA–specific daily mortality data. The number of mortalities associated with heatwaves was determined using the attributable risk percentage derived from RR estimates. The calculation incorporates the average daily number of anticipated deaths within the community and the number of days classified as heatwaves. Subsequently, the resulting value is multiplied by the attributable risk percentage. Equation (2) was applied to determine the cumulative attributable mortality (number per year):^[^ ^47^ ^]^

(4) $$Attributable\;mortality=heatwave\;days\times death_{daily}\times \frac{\left(RR-1\right)}{RR}$$

here, *death_daily_*, and *RR* refer to the annual number of days classified as heatwaves (number per year), the daily community death count (number per year), and the relative risk of heatwave effects on mortality, obtained from epidemiological studies, respectively.

Data regarding country‐specific mortality rates and population from 1950 to 2100 were acquired from the data provided by the United Nations (https://data.un.org), which projects population and demographic trends until 2100. Annual total deaths for each country were converted into daily mortality rates by dividing by 365 (or 366 for leap years). Due to the unavailability of daily mortality data in CA, we approximated daily mortality rates by dividing the annual totals by the number of days in a year (365 or 366). evenly distributing annual totals across the year. This method simplification introduces potential biases since it averages mortality without accounting for daily fluctuations. We recognize that mortality can spike during temperature extremes, and our method could lead to both over‐ and underestimations.

No epidemiological research regarding the effects of heatwaves on human health and death have mortality has been undertaken in CA. Consequently, we employed meta‐analysis studies to derive RR values. Meta‐analyses aggregate data from various research, yielding a thorough and statistically reliable risk estimation.^[^ ^48^, ^49^ ^]^ Nonetheless, disparities in climate adaption capabilities, healthcare systems, demographic characteristics, and urbanization levels may affect mortality responses differentially between locations, hence introducing possible uncertainty in our estimations.^[^ ^48^, ^49^ ^]^ The selected relative risk for heatwave number, defined based on maximum daily temperature was 1.20 (95% CI: 1.02, 1.41). We adopted a pooled RR value reported in multi‐country meta‐analyses, which provide statistically robust estimates of excess mortality risk during heatwaves. The selected pooled RR was 1.20 (95% CI: 1.02–1.41) for all‐cause mortality during heatwave days defined by daily maximum temperature thresholds.^[^ ^48^, ^49^ ^]^ This RR was applied uniformly across CA countries to compute attributable fractions and heatwave‐related deaths.

We acknowledge that this approach differs from the original Nori–Sarma et al.^[^ ^47^ ^]^ implementation, which combined propensity score matching with quasi‐Poisson regression, which applied propensity score matching with daily mortality data, as well as from the widely used Distributed Lag Non‐Linear Model (DLNM) framework.^[^ ^50^ ^]^ Both require methods require detailed daily mortality and temperature records, which are not available for CA. Given these data constraints, the application of pooled RR values provides a pragmatic and policy‐relevant first‐order estimate of heatwave‐attributable mortality, while introducing uncertainties related to differences in climate adaptation, healthcare systems, demographics, and urbanization levels across regions.

## 3 Results

### 3.1 Statistical Assessment of Bias Correction Performance

**Table** **2** and Tables S3–S7 (Supporting Information) presents the statistical evaluation of multi‐ensemble GCMs of T_max_ and T_min_ for each month in CA from 1959 to 2021. The corrected multi‐ensemble GCM models of T_max_ exhibit a lower magnitude of bias and RMSE compared to uncorrected models during the warm period of the year (May–September) relative to observations across all included stations in CA. This indicates a closer match in interannual T_max_ variation to the observations. However, they did not demonstrate any improvement in terms of interannual T_min_ variation relative to observations during the warm period of the year (Table S3, Supporting Information) by overestimating it across CA.

**Table 2:** The number of all‐cause mortalities associated with heatwaves across CA from 1959 to 2100.

Historical (1959–2021)						
Number of all‐cause mortalities associated with HWN	Kazakhstan	Kyrgyzstan	Tajikistan	Turkmenistan	Uzbekistan	Central Asia
	3138	675	474	769	2304	7360

Near future (2022–2051)						
SSP2–4.5	1909	446	400	536	989	4280
SSP5–8.5	2314	593	563	574	2003	6047

Far future (2071–2100)						
SSP2–4.5	2284	810	1063	855	2383	7395
SSP5–8.5	4066	1659	1265	1203	4034	12 227

Conversely, the corrected multi‐ensemble GCM models were able to closely replicate the overall observed interannual T_min_ variation pattern during the cold period, contrasting with their performance in capturing the variation in T_max_ during the cold period. The corrected models exhibited a similar pattern in all countries individually as well (Tables S4–S8, Supporting Information). Given the reasonable improvement in multi‐ensemble GCM models, we decided to utilize the heatwave definition based on maximum temperature in calculating all heatwave‐related indices, rather than those proposed based on minimum temperature and excess heat factor. This decision aimed to provide a more accurate depiction of future projections. Further investigations to improve variable of interest forecasts using different bias correction methods, such as LSTM machine learning model, is recommended.^[^ ^46^, ^51^ ^]^

### 3.2 Spatial and Temporal Variations in the Climate Indices in CA

#### 3.2.1 Changing Patterns in the Percentage of Warm Days and Nights

**Figure 2:**
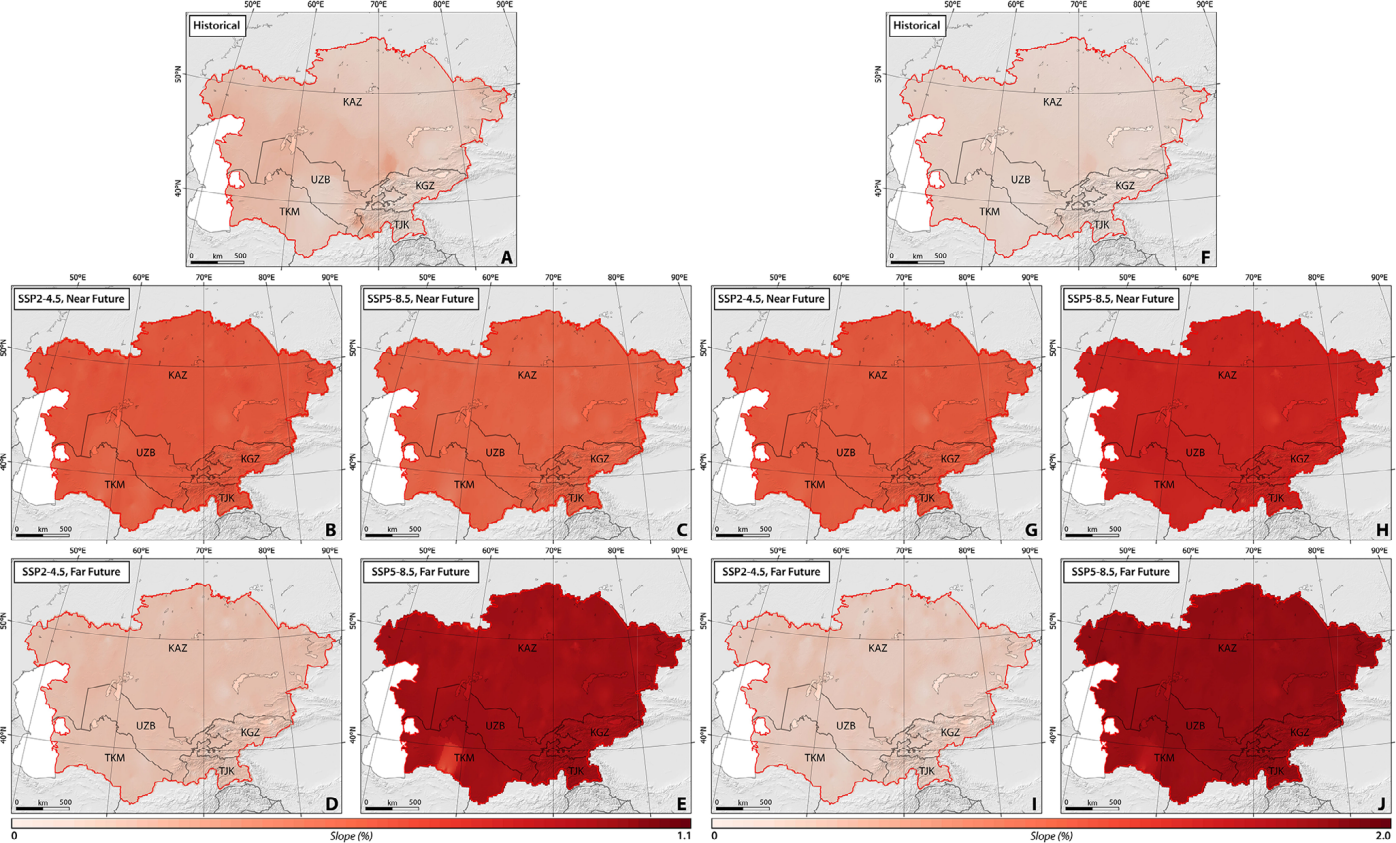
The slope of spatial‐temporal changes in the percentage of warm days (TX90p) on the left side and warm nights (TN90p) on the right side across CA, considering SSP2–4.5 and SSP5–8.5 climate projections between 1959 and 2100.

To analyze past and projected heatwave‐related indices, we examined the statistical significance of both historical and future warm days (TX90p) and nights (TN90p). **Figure** **2** illustrates the spatial and temporal distribution of the proportion of warm days and nights. Table S9 (Supporting Information) shows the statistics of calculated climate indices separately in the studied countries between 1959 and 2100 under both climate scenarios. Our findings reveal a statistically significant rise in the proportion of warm days and warm nights, with their highest values recorded in Taraz (Kazakhstan) with values of 0.375, and 0.378, respectively, in CA (Figure 2). The proportion of warm days and warm nights range from 0.30 to 44.4%, and 0.30–43.0%, respectively, in CA between 1959 and 2021 (Table S9, Supporting Information).

Analyzing the trends in TN90p and TX90p across the map has enabled the distinction of CA into two distinct halves based on the extent of climate change. The northern and southwestern regions exhibit a significant trend toward elevated extreme daytime and nighttime temperatures. In contrast, the northeastern and southern portions demonstrate a comparatively less pronounced trend, both in terms of extremely high nighttime temperatures and elevated daytime temperatures.

Future projections suggested that both TX90p and TN90p will experience a significant spike (*p*‐value < 0.05) in the near future under both studied scenarios. Under SSP2–4.5, projections indicate that TX90p and TN90p are expected to rise by slopes ranging from 0.30 to 0.70 and 0.80 to 1.20, respectively. In the far future, TX90p and TN90p are projected to increase by smaller slopes ranging from 0.10 to 0.30 and 0.10 to 0.40, respectively. The proportion of warm days and nights is estimated to range from 2.0 to 34.3% and 0.50 to 54.0%, respectively, in Central Asia (CA) between 2022 and 2051. Conversely, between 2071 and 2100, the proportion of warm days and nights is anticipated to range from 0.30 to 22.13% and 4.1 to 28.0%, respectively, in CA.

SSP5–8.5 projected comparable shifts in TX90p, varying from 0.30% to 57.30%, and greater variations in TN90p, spanning from 1.10% to 57.30%, in the near future. Looking further ahead, significant rises in the percentage of warm days and nights are anticipated under SSP5–8.5, with projections ranging from 1% to 35.3% and from 0.30% to 59.3%, respectively, in the far future.

It is important to note that due to the overestimation of interannual T_min_ variations relative to observations in multi‐ensemble GCM models (as discussed in Section 3.1), there may be an overestimation in the percentage of warm nights throughout the research period in CA.

#### 3.2.2 Changing Patterns in Heatwaves

**Figure 3:**
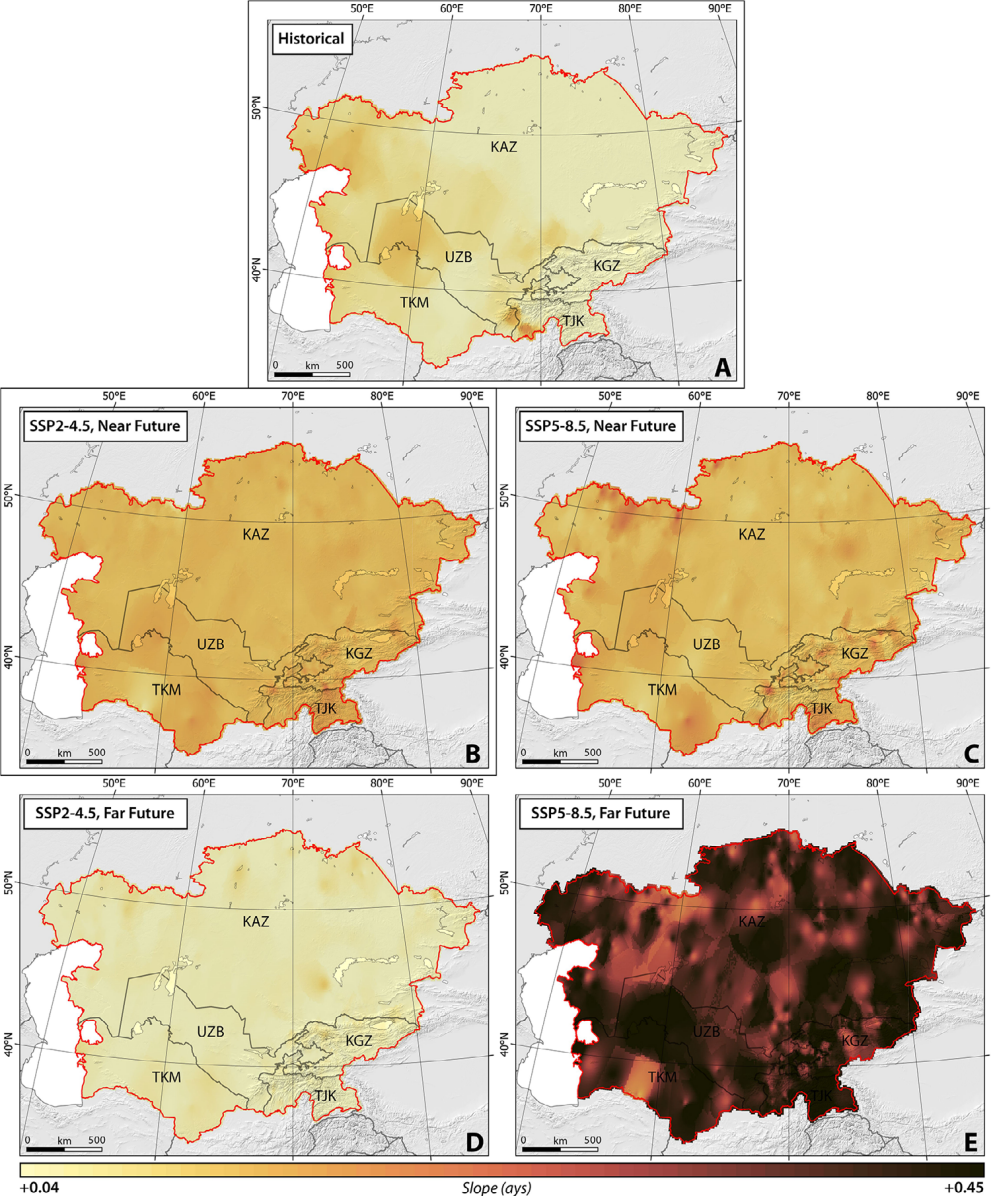
The slope of spatial‐temporal changes in HWD across CA, considering SSP2–4.5 and SSP5–8.5 climate projections between 1959 and 2100.

**Figure 4:**
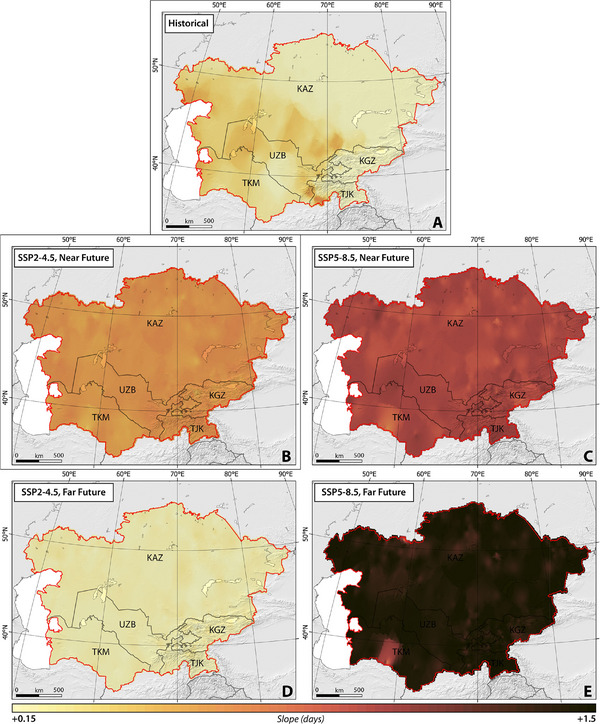
The slope of spatial‐temporal changes in HWF across CA, considering SSP2–4.5 and SSP5–8.5 climate projections between 1959 and 2100.

**Figure 5:**
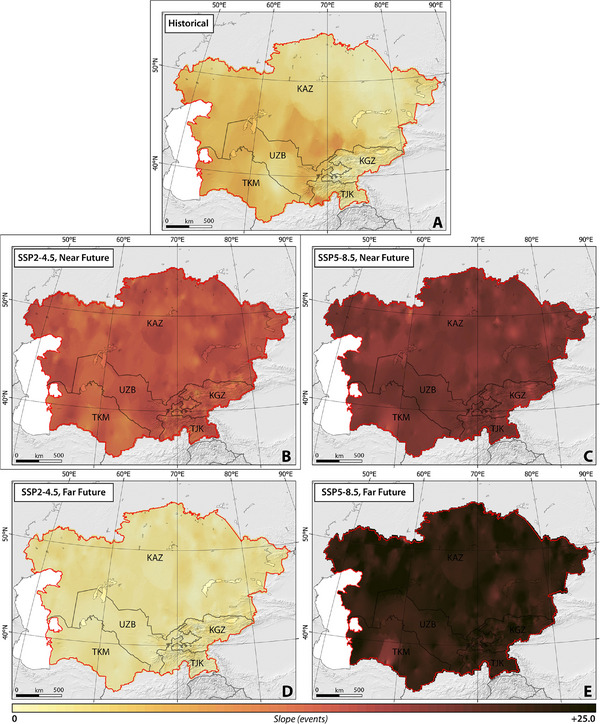
The slope of spatial‐temporal changes in HWN across CA, considering SSP2–4.5 and SSP5–8.5 climate projections between 1959 and 2100.

**Figure 6:**
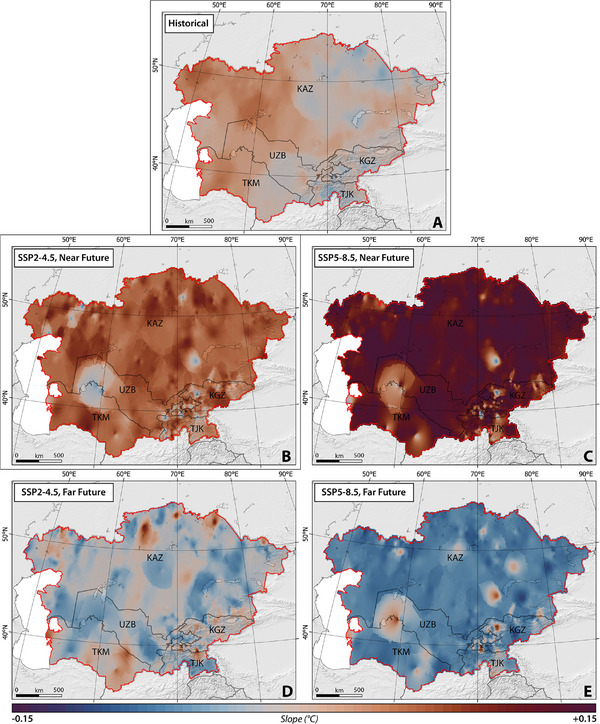
The slope of spatial‐temporal changes in HWM across CA, considering SSP2–4.5 and SSP5–8.5 climate projections between 1959 and 2100.

**Figure 7:**
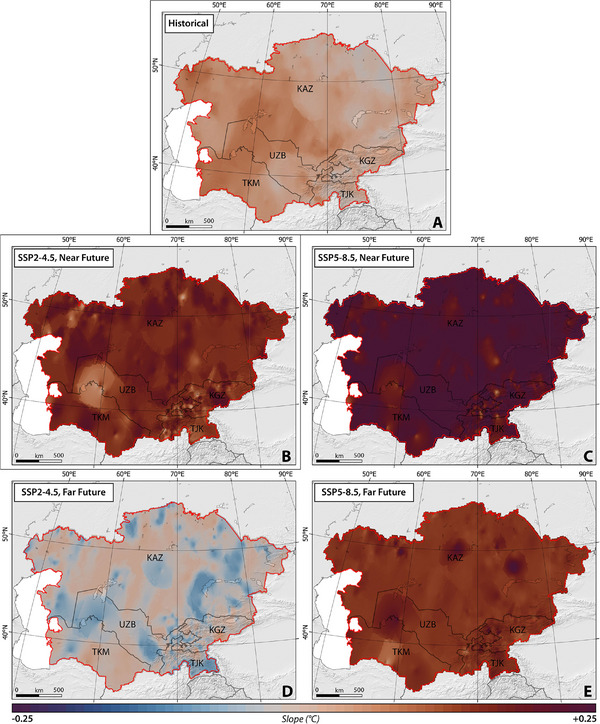
The slope of spatial‐temporal changes in HWA across CA, considering SSP2–4.5 and SSP5–8.5 climate projections between 1959 and 2100.

**Figures** **3**, **4**, **5**, **6**, **7** illustrate the spatial and temporal distribution of HWD, HWF, HWN, HWM, and HWA under SSP2–4.5, and SSP5–8.5 between 1959 and 2100 (historical period (1959–2021), near future (2022–2051), and far future (2071–2100)).

##### HWD, HWF, and HWN

Our findings suggested that the western regions of Kazakhstan, as well as nearly the entirety of Uzbekistan and Turkmenistan, exhibited pronounced susceptibility to rapid changes, particularly increases, in HWD, HWF, and HWN across CA from 1959 to 2021 (Figures 3, 4, 5). Turkmenistan demonstrated the highest slopes for HWD, Uzbekistan for HWF, and Kyrgyzstan for HWN, with average values of 0.07, 0.32, and 0.17, respectively.

Under the SSP2–4.5 scenario, the modeled HWD, HWF, and HWN over a 30‐year climatic period (near future) exhibit a statistically significant increase in the slope of changes across almost all CA, as depicted in Figures 3, 4, 5. The rate of change is relatively consistent across the studied countries, ranging from 0.14 ± 0.01 (Kazakhstan) to 0.15 ± 0.02 (Uzbekistan) for HWD, 0.60 ± 0.07 (Turkmenistan) to 0.70 ± 0.05 (Kazakhstan) for HWF, and 0.12 ± 0.02 (Turkmenistan) to 0.14 ± 0.01 (Kazakhstan) for HWN, respectively. Conversely, under SSP5–8.5, sharper changes in HWF and HWN are projected across the entirety of CA, although changes in HWD are not uniformly sharp. Certain regions, such as the northeast and southwest of Kazakhstan, the south and east of Turkmenistan, northeast Tajikistan, and northwest Kyrgyzstan, demonstrate higher vulnerability to increases. The rate of change is estimated to range from 0.05 ± 0.01 (Kazakhstan) to 0.06 ± 0.02 (Turkmenistan) for HWD, 0.20 ± 0.03 (Uzbekistan) to 0.21 ± 0.05 (Kyrgyzstan) for HWF, and 0.01 ± 0.01 (Uzbekistan) to 0.02 ± 0.01 (Kazakhstan) for HWN, respectively.

In the far future, under the SSP2–4.5 scenario, the modeled average HWD, HWF, and HWN exhibit differing projections, with a reduction in the rate of change compared to the near future. However, a statistically significant increasing trend is still estimated. Meanwhile, under SSP5–8.5, there is a sharp increase in the rate of change, which is projected to be statistically significant (Figures 3, 4, 5). Similar to the near future, the rate of changes remains consistent among different countries, ranging from 0.13 ± 0.02 (Kazakhstan) to 0.14 ± 0.04 (Tajikistan) for HWD, 0.98 ± 0.10 (Turkmenistan) to 1.02 ± 0.07 (Uzbekistan) for HWF, and 0.18 ± 0.02 (Turkmenistan) to 0.20 ± 0.01 (Uzbekistan) for HWN, respectively. Conversely, under SSP5–8.5, the projected sharper changes range from 0.40 ± 0.2 (Turkmenistan) to 0.42 ± 0.07 (Tajikistan) for HWD, 1.4 ± 0.20 (Turkmenistan) to 1.50 ± 0.05 (Uzbekistan) for HWF, and 0.22 ± 0.03 (Turkmenistan) to 0.23 ± 0.02 (Kazakhstan) for HWN, respectively.

Our findings suggest that across CA, both climate scenarios project an increase in HWN, HWD, and HWF in the far future in comparison to the near future, except for a projected decrease of 10% in HWNs in Kazakhstan (Table S9, Supporting Information). Analysis in Table S9 (Supporting Information) reveals that among the studied countries, Turkmenistan (under SSP2–4.5) is expected to experience the highest percentage increase, with values of 145%, 183%, and 162% in HWN, HWD, and HWF, respectively, during the far future in comparison to the near future, followed by Uzbekistan with percentages of 116%, 156%, and 132% in HWN, HWD, and HWF, respectively. Similarly, under SSP5–8.5, Turkmenistan and Uzbekistan are anticipated to encounter more frequent and prolonged heatwaves in the far future in comparison to the near future (Table S9, Supporting Information). Our analysis suggests that while climate indices are expected to rise, with a slower rate of changes, between 2071 and 2100 (Figures 3, 4, 5).

##### HWM and HWA

Throughout the historical period, findings on the magnitude of heatwaves have revealed a statistically significant increase moving from the western regions of CA toward the eastern and southern areas. Conversely, the amplitude of heatwaves has exhibited a distinct pattern, showing an overall upward trend across all of CA, except for certain areas including northeast Kazakhstan, south Kyrgyzstan, south Tajikistan, southwest Uzbekistan, and east Turkmenistan (Figures 6,7). Notably, Turkmenistan stands out with the highest slopes of HWM and HWA, averaging at 0.02 and 0.06, respectively.

The SSP2–4.5 scenario forecasts a significant, nearly uniform increase (*p*‐value < 0.05) in both the modeled mean HWM and HWA averaged over the near future across almost the entirety of CA. However, certain regions, primarily located in Kazakhstan and Uzbekistan, show resistance to these increases in HWM and HWA (Figures 6,7). The rate of change is relatively consistent across the studied countries, ranging from 0.06 ± 0.03 (Kazakhstan) to 0.04 ± 0.05 (Tajikistan) for HWM, and 0.20 ± 0.03 (Kazakhstan) to 0.13 ± 0.05 (Tajikistan) for HWA. In contrast, the SSP5–8.5 scenario projects extreme changes over time in both HWM and HWA across the entire CA region, with rates ranging from 0.13 ± 0.03 (Kazakhstan) to 0.10 ± 0.08 (Turkmenistan) for HWM, and 0.23 ± 0.03 (Kazakhstan) to 0.20 ± 0.07 (Turkmenistan) for HWA.

Spanning 2071 to 2100, under the SSP2–4.5 scenario, it is projected that the modeled mean HWM and HWA will decrease (*p*‐value < 0.05). In addition, the SSP5–8.5 scenario projected a sharper reduction in the rate of change (*p*‐value < 0.05). Notably, while the slope of changes in HWA remains positive (indicating an increase but at a lower rate) under SSP5–8.5, HWM is expected to decrease sharply in CA (Figures 6 and 7). The forecasted rates of changes in HWM and HWA range from −0.02 ± 0.03 (Kazakhstan) to −0.01 ± 0.05 (Uzbekistan), and −0.03 ± 0.05 (Tajikistan) to −0.01 ± 0.05 (Turkmenistan), respectively, under SSP2–4.5.

In contrast, within the SSP5–8.5 scenario, more pronounced alterations are anticipated, with variations ranging from −0.06 ± 0.02 (Kazakhstan) to −0.04 ± 0.05 (Turkmenistan) for HWM, and from 0.13 ± 0.04 (Turkmenistan) to 0.15 ± 0.03 (Kyrgyzstan) for HWA.

According to our findings, an increase is expected in both the magnitude and amplitude of heatwaves in the far future relative to the near future across CA under both climate scenarios (Table S9, Supporting Information). Among the countries studied, Turkmenistan is projected to experience the highest percentage increases under SSP2–4.5, with values of 122% and 120% in HWM and HWA, respectively, during the far future in comparison to the near future. Uzbekistan follows closely behind with percentages of 114% and 123% in HWM and HWA, respectively. Under SSP5–8.5, Kazakhstan and Kyrgyzstan are expected to experience higher degrees of HWM and HWA in the far future in comparison to the near future (Table S9, Supporting Information). Despite the decreasing trend in the changes of magnitude and amplitude of heatwaves, our results suggest that their values will still be higher than the projected degrees in the near future. This implies that both HWM and HWA may increase in the mid‐future in comparison to the near future and then begin to decrease during the far future under both climate scenarios (Figures 6 and 7).

We conducted a classification of the annual HWM in CA between 1959 and 2021 (Table 1). Notably, the lower tail of the normal category was observed in Kazakhstan, Tajikistan, and Kyrgyzstan, while the upper tail was evident in Uzbekistan and Turkmenistan (Table 1). Under SSP2–4.5, there is an anticipated shift in the normal category toward higher values in HWM during both the near and far futures, ranging from 26 to 31 °C. Comparing these future projections to the historical period, it can be inferred that future normal heatwaves will be comparable to moderate and severe heatwaves experienced during the historical period in terms of HWM.

For SSP5–8.5, the shift in the normal category is expected to be even higher, ranging from 29 to 36 °C. The prevalence of higher tails remains dominant in Turkmenistan and Uzbekistan in the future. However, it is worth noting that super extreme and ultra‐extreme events were not encountered during the historical period in CA. Future projections indicate the likelihood of super extreme heatwaves occurring mainly in Kazakhstan, Turkmenistan, and Tajikistan under both climate scenarios. Furthermore, it is anticipated that Turkmenistan may experience ultra‐extreme heatwaves in the far future. Apart from Turkmenistan, none of the other countries is expected to undergo ultra‐extreme events in CA in the future.

#### 3.2.3 Health Risk Assessment Associated with Heatwaves

**Figure 8:**
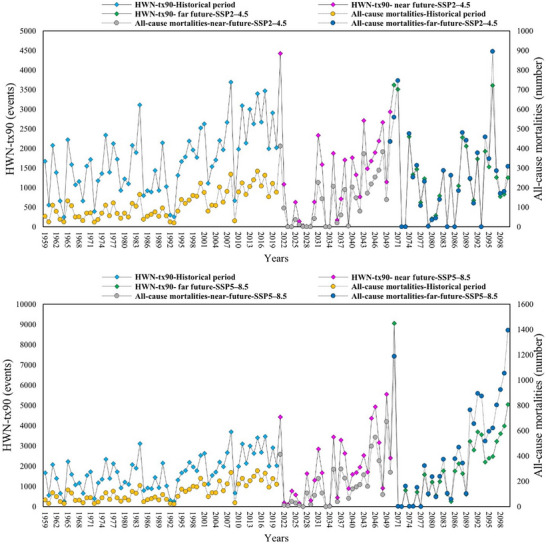
The temporal distribution of HWN and all‐cause mortalities considering (A) SSP2–4.5 and (B) SSP5–8.5 climate projections between 1959 and 2100 between across CA.

Building on the projected intensification of heatwave indices, we assessed the associated health risks by estimating all‐cause mortality under SSP2–4.5 and SSP5–8.5 for the historical (1959–2021), near future (2022–2051), and far future (2071–2100) periods. The results reveal the temporal evolution of heatwave‐related mortality across CA and its five countries (Uzbekistan, Kyrgyzstan, Kazakhstan, Turkmenistan, and Tajikistan (**Figure** **8**; Figures S1–S5, Supporting Information).

##### Historical Period

Our findings reveal a consistent and statistically notable increase (*p*‐value < 0.05) in all‐cause mortalities across CA (Table S10, Supporting Information). During the historical period, Kazakhstan exhibited the highest statistically notable increments (*p*‐value < 0.05) in the number of mortalities compared to other countries, followed by Uzbekistan, while Kyrgyzstan experienced the lowest changes (Table S10, Supporting Information). The slope of changes was calculated to be 1.267 and 1.078 in Kazakhstan and Uzbekistan, respectively, whereas Kyrgyzstan demonstrated a slope of change of 0.172 (Table S10, Supporting Information). Furthermore, Table 2 provide insights into the health outcomes associated with heatwaves, along with the total count of heatwaves observed in CA during the study period. It is worth noting that the distribution of locations and their demographic data is not uniform across different countries.

Consequently, countries experiencing a higher number of heatwaves may not necessarily exhibit a higher number of mortalities due to lower population densities. Kazakhstan and Uzbekistan demonstrated the highest estimated mortality counts, which did not align with the number of heatwaves across these countries from 1959 to 2021 (Table 2; Table S11, Supporting Information). Conversely, Tajikistan and Kyrgyzstan reported comparatively lower associated mortality figures, despite experiencing a higher number of heat waves following Kazakhstan (Table 2). According to our findings, CA experienced its highest number of heatwaves in the years 1984 and 2008, with counts of 3110 and 3695, respectively, between 1959 and 2021 (Figure 8). Consequently, the all‐cause mortalities followed an increasing trend in line with the number of heatwaves, reaching peaks of 165 and 268 in 1984 and 2008, respectively, across CA.

##### Future Projections

In line with the increasing trend of all‐cause mortalities in CA from 1959 to 2021, future projections under both climate scenarios indicate a statistically notable rise in the number of deaths corresponding to heatwaves in all countries during the near and far futures (excluding all‐cause mortalities in the far future) (Table S10, Supporting Information). Under the SSP2–4.5 scenario, Kazakhstan, followed by Uzbekistan, is projected to experience a higher increasing rate in all‐cause mortalities associated with HWN, with slopes of 5.432 and 3.021, respectively, in the near future. A similar increasing trend is anticipated in the far future, but with different slopes of 1.377 and 1.102 for Kazakhstan and Uzbekistan, respectively (Table S10, Supporting Information). The SSP5–8.5 scenario yields comparable results and trend of changes in both the near and far futures, but with higher values (higher slopes of changes compared to SSP2–4.5).

Despite Kazakhstan and Uzbekistan showing a higher tendency for an increase in all‐cause mortalities, Turkmenistan demonstrates lower vulnerability to heatwave‐related mortalities in both the near and far futures under both scenarios (except in the near future under SSP2–4.5, where Kyrgyzstan appears susceptible to the lowest rate of death).

In the near future, the number of heatwaves exhibits a spike in the beginning, with 2022 recording a value of 4425. Although there are some fluctuations, there is an overall increasing trend over time, reaching a peak in 2051 with 3618 events under SSP2–4.5. Conversely, under SSP5–8.5, there is no initial spike in 2022, but the trend of changes remains upward, with the number of heatwaves mounting to 9056 in 2051. Similar temporal changes are projected for the number of heatwaves in the far future under both scenarios (Figure 8).

Our findings suggest a lower slope of changes in the far future in comparison to the near future, likely due to variations in projected heatwave occurrences stemming from factors such as climate change mitigation strategies, adaptation measures, uncertainties in climate models, and natural fluctuations in the climate system.

## 4 Discussion

An ensemble of CA regional climate projections was investigated under SSP2–4.5 and SSP5–8.5. As there is no universally accepted definition of heatwaves, we applied the standard 90th percentile of daily maximum temperature (T_max_) across all indices and adopted the HWM index, which integrates both intensity and duration, to enable clearer severity interpretation and regional comparison.^[^ ^38^, ^39^, ^40^ ^]^

Previous results are consistent with our findings and support our multi‐model assessment for CA, suggesting that there will be a shift toward extremely hot weather later in the 21st century under both climate scenarios in comparison to the historical period. This will include unprecedented occurrences of ultra‐ and super‐extreme heatwaves, which are expected to emerge by the middle of the century and could become regular summertime conditions later in the 21st century.^[^ ^9^, ^16^, ^18^, ^19^ ^]^

The pronounced emergence of super‐ and ultra‐extreme heatwaves in Turkmenistan can be attributed to its predominantly arid lowland environment, including the vast Karakum Desert, where limited soil moisture amplifies land–atmosphere feedback and intensifies near‐surface warming. Such soil moisture–temperature coupling has been identified as a critical driver of heatwave amplification in arid and semi‐arid regions.^[^ ^5^, ^11^ ^]^ Additionally, persistent anticyclonic high‐pressure systems enhance subsidence and radiative heating, further reinforcing extreme heat.^[^ ^52^ ^]^ In contrast, the orographic complexity and higher moisture availability in Kyrgyzstan and Tajikistan moderate extreme heatwave development, consistent with prior findings on topographic and hydrological buffering of heat extremes.^[^ ^53^ ^]^ These mechanisms explain the spatial heterogeneity in projected heatwave intensification across CA.

What distinguishes our study from earlier CMIP6‐based analyses is that we provide one of the first region‐wide, high‐resolution assessments of heatwaves in CA using 700 stations, apply multiple heatwave indices, and directly link climate projections to all‐cause mortality estimates. This integrated climate‐health perspective is novel for the region and extends beyond previous work that has mainly concentrated on mean temperature or precipitation trends.^[^ ^7^, ^17^, ^22^ ^]^

Our results suggest that in certain areas of CA, peak temperatures during future heatwaves could surpass 35 °C, which in counties of Tajikistan and Kyrgyzstan is 133% and 150% times higher compared to base period, respectively (Table S9, Supporting Information). These conditions pose a significant risk to human life and even livestock, particularly in urban areas where the UHI effect is expected to further intensify maximum temperatures, disrupting societal functions. It is projected that urbanization will increase under both climate scenarios, suggesting that the majority of the population will reside in urban complexes that are predicted to grow in both population density and size. The projected spike in urbanization will cause an intensified UHI effect, consequently leading to increased heat stress.^[^ ^41^ ^]^ This analysis does not address the impact of the UHI effect because climate models cannot capture the details of urban areas. Besides, it is also important to consider the variability in the UHI effect, which depends on several factors such as thermal properties, orientation, the urban canopy's albedo, and local flow conditions.^[^ ^54^ ^]^

Past heatwave occurrences indicate that critical factors influencing mortality risk include the duration and intensity of extreme temperatures, as well as the rate of temperature escalation. Over the past four decades, there has been a consistent rise in summer mortality rates, suggesting an impact from warming. Heatwaves globally have been linked to devastating mass mortality events in recent years, including in Europe (70000 deaths in 2003), Russia (55000 deaths in 2010),^[^ ^8^ ^]^ and thousands in the Indian subcontinent in 2015. A recent study utilizing empirical data from 732 sites across 43 nations found that ≈37.0% of heat‐related deaths during warmer seasons can be attributed to human‐induced climate change. Increased mortality was observed across all continents.

As the severity of heatwaves increases across CA, this crisis must be recognized as both a public health emergency and a broader climate adaptation challenge. Heat‐related mortality will rise significantly under both climate scenarios, with ultra‐extreme heatwaves expected in Kazakhstan, Turkmenistan, and Tajikistan by the late 21st century. Given the projected intensification of extreme temperatures, adaptation policies must align with both climate mitigation and health system preparedness to reduce mortality and economic losses. Adaptation policies have to deal with both direct health risks (e.g., hospital admissions, heat‐related mortality) and indirect effects (e.g., water supply interruptions, increased energy demand, and reduced agricultural productivity). A coordinated approach is necessary to incorporate climate forecasts, disaster preparedness, urban heat mitigation, and early warning systems into national adaptation strategies.

In the Middle East and North Africa (MENA) region, nearly fifty percent of the population experiences annual occurrences of normal (0 °C < daily HWM < 5 °C) or moderate (5 °C < daily HWM < 10 °C) heatwaves, with millions affected by severe (10 °C < daily HWM < 15 °C) events annually.^[^ ^41^ ^]^ This figure is expected to peak ≈2065 before declining. By 2060, unprecedented super‐extreme (50 °C < daily HWM < 80 °C) and ultra‐extreme (daily HWM >80 °C) heatwaves are projected to impact over 100 million individuals, continuing to escalate in subsequent decades. Later in the 21st century, approximately fifty percent of the population, an estimated 600 million inhabitants, maybe experience such extreme events. Due to the UHI effect, particularly in megacities and urban areas, an additional 400 million people are projected to face annual instances of high‐impact heatwaves.^[^ ^41^ ^]^

Urban adaptation strategies should be focused on reducing the UHI effect, mainly in densely populated areas with projected amplified heatwave intensity. Urban heat exposure can be reduced by undertaking measures such as increasing albedo surfaces, expanding urban green spaces, and implementing heat‐resilient infrastructure.^[^ ^55^ ^]^ Conversely, rural and mountainous areas may encounter reduced intensities of heatwaves; however, they remain susceptible to adverse impacts owing to inadequate infrastructure and insufficient emergency response capabilities. Adaptation in these domains should prioritize the enhancement of early warning systems and the fortification of healthcare preparedness in order to mitigate heat‐related morbidity and mortality.^[^ ^38^, ^56^, ^57^ ^]^ Our findings suggest that the inadequate condition of health services, along with the growing frequency and severity of heatwaves, could potentially overwhelm existing adaptation strategies and underscore the need for proactive planning and investment in heat‐resilient policies in CA.^[^ ^16^, ^18^ ^]^

Comparisons with other regions also support these findings. For example, in the MENA region, historical heatwaves typically lasted 4–6 days but are projected to persist for weeks by the end of the century, with maximum temperatures surpassing 56 °C in hotspots.^[^ ^41^ ^]^ Similarly, the frequency of high‐impact events is expected to rise substantially, especially under high‐emission scenarios. These projections align with our results for CA, where heatwave duration and intensity are expected to increase markedly toward the end of the century, underscoring the broader global tendency toward longer and more intense heat extremes.

Our results correspond with research investigating the intensification of heatwaves in comparable arid and semi‐arid climates. Zittis et al.^[^ ^41^ ^]^ project that business‐as‐usual emissions will cause super and ultra‐extreme heatwaves in the MENA region by the century's end, resulting in near‐continuous heatwave conditions in metropolitan areas. Likewise, areas within the MENA are anticipated to see heatwaves enduring for almost 50% of the warm season.^[^ ^58^, ^59^ ^]^

This pattern aligns with our projections for CA, where highly populated urban areas will endure extended durations of extremely high temperatures, intensified by rapid urbanization and infrastructural constraints. Areas, especially those lacking robust climate adaption strategies, may face challenges due to the heightened occurrence of heatwaves, resulting in significant socio‐economic consequences. These findings underscore the necessity for focused mitigation methods, especially in cities that are already susceptible due to high population density and inadequate heat management systems.^[^ ^58^, ^59^ ^]^

A key limitation of this study is the absence of localized epidemiological data on heatwave‐related mortality in CA, which constrains direct regional evaluations. To address this gap, we employed a meta‐analysis methodology, utilizing current research from various countries to assess potential risks to human health. We adopted pooled relative risk (RR) values reported in published multi‐country meta‐analyses and applied them uniformly to Central Asian countries. This method approach offers significant insights but may add uncertainties, as variations in urbanization rates, healthcare systems, demographic profiles, and climate adaption adaptation capacity could differentially affect mortality responses across regions. We also acknowledge that, unlike approaches such as Nori–Sarma et al.^[^ ^47^ ^]^ or the widely used Distributed Lag Non‐Linear Model (DLNM) framework,^[^ ^50^ ^]^ our analysis could not incorporate daily mortality–temperature time‐series because such data are unavailable for CA. Future research using region‐specific health data is essential to enhance impact assessments and strengthen public health preparation for severe temperature events in CA.

Another limitation is that our analysis assumed daily mortality rates to be uniformly distributed, which may underestimate or overestimate deaths during extreme events. This simplification was necessary due to data availability, but could overlook the temporal clustering of mortality during heatwaves. Furthermore, recent evidence suggests that hospital‐based statistics may substantially underestimate heat‐related health impacts, since many individuals suffering from heat stress do not seek medical attention. For example, a survey across three Chinese cities found that fewer than one quarter of affected individuals were willing to visit hospitals, while the majority managed symptoms without formal healthcare.^[^ ^60^ ^]^ Future research using region‐specific health and behavioral data is essential to refine impact assessments and strengthen public health preparedness for severe temperature events in CA.

The resolution of the climate models employed in this study is not sufficient to explicitly capture urban‐scale processes, including the urban heat island (UHI) effect. As shown in prior work, UHIs can intensify local heatwave intensity by 2–5 °C, meaning that our projections may underestimate actual risks in major Central Asian cities such as Tashkent, Almaty, and Astana.^[^ ^61^ ^]^ Previous studies further indicate that UHIs and heatwaves can act synergistically, with UHIs amplifying especially nocturnal heat stress by 1–3 °C depending on urban form, surface moisture, and ventilation conditions.^[^ ^27^ ^]^ This synergy suggests that urban residents could face substantially higher risks than those reflected in our projections. Future work should therefore integrate high‐resolution urban climate modeling and epidemiological data, including urban canopy parameterizations, to more accurately assess combined heatwave–UHI impacts in CA.

Although this study applied a multi‐model ensemble to enhance robustness, inter‐model differences remain a key source of uncertainty. These differences often arise from variations in spatial resolution, land–atmosphere coupling strength, representation of convection and cloud processes, and parameterization of surface energy and water fluxes. For example, models with stronger soil moisture–temperature coupling tend to produce more intense heatwaves due to reduced evaporative cooling.^[^ ^11^, ^62^ ^]^ In contrast, models that differ in aerosol forcing or circulation dynamics may diverge in simulating the frequency and persistence of extremes.^[^ ^63^ ^]^ While the ensemble mean provides a more reliable projection, acknowledging these structural differences helps clarify the spread across GCMs and underscores the importance of continuous model evaluation in CA.

Another limitation is that this study only considered SSP2–4.5 and SSP5–8.5 scenarios. The exclusion of SSP1–2.6 may underestimate the avoided risks under strong mitigation, while the omission of SSP3–7.0 may slightly under‐represent intermediate–high futures characterized by regional rivalry and land‐use pressures. These constraints were primarily due to computational and data management limitations. Nevertheless, SSP2–4.5 and SSP5–8.5 together span a wide and policy‐relevant portion of the emissions trajectory space,^[^ ^12^, ^64^ ^]^ ensuring that the main conclusions regarding increasing heatwave risks in Central Asia remain robust.

Despite these limitations, our findings still provide valuable insights into the potential health risks posed by extreme heat in CA, underscoring the urgent need for proactive mitigation and adaptation measures. Comparing two future pathways, it is suggested that although the SSP2–4.5 conditions may not cause as much disruption, there will still be notable adverse impacts on public health and society. In the next few decades, it is of utmost importance to prioritize the implementation of steps to reduce the impact and adjust to the negative impacts of a changing climate. Additionally, the nations in the CA region should make necessary preparations for extremely hot summers.

## 5 Conclusion and Summary

This study provides a comprehensive assessment of heatwave trends and their health implications across CA, integrating historical observations (1959–2021) and future projections (2022–2100) under moderate (SSP2–4.5) and high‐emission (SSP5–8.5) scenarios. By employing bias‐corrected CMIP6 projections, we significantly improved the accuracy of mean maximum temperature estimates—with bias reduced by 24% and RMSE lowered by 14% across CA—ensuring a more precise representation of future climatic trends.

Heatwave characteristics are projected to intensify significantly across CA. The proportion of warm days and nights will increase substantially (*p*‐value < 0.05) under both climate scenarios, though the rate of change is slower in SSP2–4.5 than in SSP5–8.5. Our findings show that all major heatwave indices (HWD, HWF, HWN, HWM, and HWA) will experience a statistically significant rise (*p* < 0.05) across the region. The trend of change remains consistent between countries, with similar increases in HWD and HWF across CA.

By classifying HWM, we observed a distinct shift in normal heatwave intensity—from historical values of 12–23 °C to future projections of 26–31 °C, making what were previously moderate and severe heatwaves the new norm. More extreme temperature events will become increasingly prevalent, with Turkmenistan and Uzbekistan facing the highest temperature extremes. Ultra‐extreme heatwaves, which were absent in the historical period, are projected to emerge in Kazakhstan, Turkmenistan, and Tajikistan by the end of the century, presenting unprecedented risks to human health and infrastructure.

We systematically examined the relationship between heatwaves and all‐cause mortality, revealing a statistically significant increase in heatwave‐related deaths (*p* < 0.0001) in CA, both in historical data and future projections. The strongest mortality increases are expected in Kazakhstan and Uzbekistan, where heatwave frequency and duration will rise the most. Under SSP2–4.5, Kazakhstan and Uzbekistan exhibit the highest increasing rates of heatwave‐related mortality (5.432 and 3.021 per decade, respectively) in the near future, with a continued but slower rise in the far future. The rate of increase in heatwave‐related deaths accelerates under SSP5–8.5, emphasizing the pressing need for mitigation strategies.

The trend of changes in mortalities and heatwave occurrences align, even though countries with a higher mortality rate do not necessarily correspond to higher numbers of heatwaves. This discrepancy likely arises from differences in population distribution, age demographics, healthcare infrastructure, and socio‐economic resilience. Future projections indicate that more intense and prolonged heatwaves will disproportionately impact densely populated urban centers, exacerbating the UHI effect, particularly in densely populated areas.

The escalation of heatwave intensity across CA, compounded by urbanization and climate change, necessitates targeted adaptation strategies to mitigate human health risks, urban overheating, and infrastructure vulnerabilities. Our findings indicate that densely populated cities will experience heightened UHI effects, exacerbating heat‐related mortality risks. Additionally, low green space per capita in urban areas (7.6 vs 18.2 m^2^ in Europe) further intensifies urban heat exposure, making heat adaptation an urgent priority.^[^ ^65^ ^]^

In light of these findings, several actionable strategies emerge that can directly inform policy and practice: the development and enforcement of national and regional heat action plans; investments in climate‐responsive healthcare systems with special focus on vulnerable groups such as the elderly, children, and outdoor workers; incorporation of urban heat island mitigation into city planning through greening, reflective materials, and cooling shelters; and strengthened cross‐border cooperation to share data, forecasts, and best practices.

Given the accelerating frequency, intensity, and health impacts of heatwaves across CA, immediate action is required to adapt infrastructure, improve public health resilience, and implement robust climate policies. Our findings underscore the urgent need for targeted adaptation measures, particularly in densely populated cities and vulnerable rural communities, to mitigate the devastating effects of future heatwaves in CA.

## Conflict of Interest

The authors declare no conflict of interest.

## Author Contributions

P.B. contributed to writing (review and editing), conceptualization, methodology, validation, formal analysis, investigation, data curation, and data analysis. M.B. contributed to methodology, data curation, conceptualization, validation, review and editing, resources, project administration, and funding acquisition. A.M.F. contributed to methodology, formal analysis, data curation, investigation, and data analysis. M.H., M.A., and A.R. contributed to formal analysis, data curation, and data analysis. A.F. contributed to formal analysis, data curation, and data analysis. S.S. contributed to formal analysis and data curation. M.L. contributed to supervision, investigation, and review and editing. P.K. contributed to review and editing and supervision. J.R.K. contributed to review and editing, resources, data curation, project administration, and funding acquisition. The work was supported by the Advanced Research Opportunities Program (AROP) of the RWTH Aachen University.

## Supporting information



Supporting Information

## Data Availability

The data that support the findings of this study are available from the corresponding author upon reasonable request.
