# Supplementary material for: The Escalating Threat of Heatwaves in Central Asia: Climate Change Impacts and Public Health Risks
Source: Glob Chall. 2025 Nov 8;9(12):e00401. doi: 10.1002/gch2.202500401 (PMC12697080; doi:10.1002/gch2.202500401)
Supplement: Supplementary file 1 — Supporting Information [file GCH2-9-e00401-s001.docx]

**The Escalating Threat of Heatwaves in Central Asia: Climate Change Impacts and Public Health Risks**

Parya Broomandi^1,2^, Mehdi Bagheri^2*^, Ali Mozhdehi Fard^1^, Mostafa Hadei^3,4^, Mohammad Abdoli^5^, Adib Roshani^6^, Aram Fathian^7^, Sadjad Shafiei^2^, Michael Leuchner^5^, Prashant Kumar^8,9^, Jong Ryeol Kim^1*^

^1^ Department of Civil and Environmental Engineering, School of Engineering and Digital Sciences, Nazarbayev University, Kabanbay Batyr Ave. 53, Astana 010000 Kazakhstan.

^2^Department of Electrical and Computer Engineering, School of Engineering and Digital Sciences, Nazarbayev University, Kabanbay Batyr Ave. 53, Astana 010000 Kazakhstan.

^3^Department of Health in Emergencies and Disasters, Tehran University of Medical Sciences, Tehran, Iran.

^4^Climate Change and Health Research Center (CCHRC), Institute for Environmental Research (IER), Tehran University of Medical Sciences, Tehran, Iran

^5^Physical Geography and Climatology, Department of Geography, RWTH Aachen University, Wüllnerstr. 5b, 52056 Aachen, Germany.

^6^Faculty of Civil Engineering, Babol Noshirvani University of Technology, Iran.

^7^Water, Sediment, Hazards, and Earth-surface Dynamics (waterSHED) Lab, Department of Geoscience, University of Calgary, Canada.

^8^Global Centre for Clean Air Research (GCARE), School of Sustainability, Civil and Environmental Engineering, Faculty of Engineering and Physical Sciences, University of Surrey, Guildford GU2 7XH, Surrey, United Kingdom.

^9^Institute for Sustainability, University of Surrey, Guildford GU2 7XH, Surrey, United Kingdom.

* Corresponding authors. Email: jong.kim@nu.edu.kz, and Phone: +7 (7172) 70-91-36.

Email: mehdi.bagheri@nu.edu.kz, and Phone: +7 (7172) 70-92-51.

| **Table S1.** The list of GCM models used in the current study ([https://www.ipcc.ch/report/ar6](https://www.ipcc.ch/report/ar6/wg1/downloads/report/IPCC_AR6_WGI_AnnexII.pdf)). | | |
| --- | --- | --- |
| MODELS | Resolution(km) and Number of Levels (L) | Institution/Country |
| ACCESS-CM2 | 140 km, 85 L | csiro-access csiro and Australia. res. council center of excellence for climate system science, Australia |
| BCC-CSM2-MR | 100 km, 46 L | BCC, Beijing Climate Centre, China |
| CESM2 | 100 km | NCAR, National Center for Atmospheric Research, USA |
| FGOALS-f3-L | 90 km, 32 L | CAS, Chinese Academy of Sciences, China |
| GFDL-ESM4 | 100 km, 49 L | NOAA-GFDL, National Oceanic and Atmospheric Administration, Geophysical Fluid Dynamics Laboratory, USA |
| IPSL-CM6A-LR | 160 km, 79 L | IPSL, Institute Pierre- Simon Laplace, France |
| MIROC6 | 250 km, 40 L | MIROC Consortium JAMSTEC, AORI, NIES, R-CCS, Japan |
| MIROC-ES2L | 250 km, 40 L | MIROC Consortium JAMSTEC, AORI, NIES, R-CCS, Japan |
| MPI-ESM1-2-LR | 170 km, 47 L | MPI-M, Max Planck Institute for Meteorology, Germany |
| MRI-ESM2-0 | 100 km, 80 L | MRI, Meteorological Research Institute, Japan |

| **Table S2.** Climate indexes with their definitions and units used in the current study. | | | | |
| --- | --- | --- | --- | --- |
| **ID** | **Index Name** | **Definition** | **Unit** | **Sectors of Economics** |
| HWD-Tx90 | Heatwave duration as defined by 90^th^ percentile of TX. | The length of the longest heatwave identified by HWN. | days | Health, Agriculture and Food Security, Water Resources and Hydrology |
| HWA-Tx90 | Heatwave amplitude as defined by the 90^th^ percentile of TX. | The peak daily value in the hottest heatwave (defined as the heatwave with highest HWM). | °C | Health, Agriculture and Food Security, Water Resources and Hydrology |
| HWF-Tx90 | Heatwave frequency as defined by the 90^th^ percentile of TX. | The number of days that contribute to heatwaves as identified by HWN. | days | Health, Agriculture and Food Security, Water Resources and Hydrology |
| HWN-Tx90 | Heatwave number as defined by 90^th^ percentile of TX. | The number of individual heatwaves that occur each summer (Nov – Mar in southern hemisphere and May – Sep in northern hemisphere). | events | Health, Agriculture and Food Security, Water Resources and Hydrology |
| HWM-Tx90 | Heatwave magnitude as defined by the 90^th^ percentile of TX. | The mean temperature of all heatwaves identified by HWN. | °C | Health, Agriculture and Food Security, Water Resources and Hydrology |
| TX90p | Amount of hot days. | Percentage of days when TX > 90th percentile. | % | Energy |
| TN90p | Amount of warm nights. | Percentage of days when TN > 90th percentile. | % | Energy |

### **Heat waves’ indices**

All the heatwave-related indices are calculated based on T_max_ (maximum temperature explained below:

- CTX90pct: The threshold is the 90^th^ percentile of T_max_ (maximum temperature) for each calendar day, considering a 15-day window. This means there is a unique percentile value for each day of the year, accounting for seasonal variations, with the window centered on the respective day. The use of a moving window ensures temporal dependence is considered while obtaining a sufficient sample size to determine a realistic percentile value. The thresholds are calculated separately for each period and grid box.

|  | **Table S3.** The statistical assessment of multi-ensembled GCM models of T_max_ and T_min_ for each month across CA between 1959 and 2021. | | | | | | | | | | | | |
| --- | --- | --- | --- | --- | --- | --- | --- | --- | --- | --- | --- | --- | --- |
| **Tmax** | **Raw multi-ensembled GCM models** | | | **Bias-corrected multi-ensembled GCM models** | | | **Tmin** | **Raw multi-ensembled GCM models** | | | **Bias-corrected multi-ensembled GCM models** | | |
|  | **Month** | **Bias** | **RMSE** | **Month** | **Bias** | **RMSE** |  | **Month** | **Bias** | **RMSE** | **Month** | **Bias** | **RMSE** |
|  | ***January*** | -12.42 | 17.63 | ***January*** | -15.40 | 19.43 |  | ***January*** | -20.07 | 23.63 | ***January*** | -15.52 | 19.49 |
|  | ***February*** | -12.41 | 17.45 | ***February*** | -15.37 | 19.27 |  | ***February*** | -20.82 | 24.10 | ***February*** | -16.30 | 19.93 |
|  | ***March*** | -8.57 | 15.03 | ***March*** | -11.49 | 16.21 |  | ***March*** | -17.23 | 21.05 | ***March*** | -11.95 | 16.46 |
|  | ***April*** | -1.90 | 12.96 | ***April*** | -4.77 | 12.34 |  | ***April*** | -10.91 | 16.44 | ***April*** | -4.54 | 12.14 |
|  | ***May*** | **5.90** | **14.28** | ***May*** | **3.02** | **11.82** |  | ***May*** | **-4.08** | **13.35** | ***May*** | **3.41** | **11.71** |
|  | ***June*** | **13.10** | **18.42** | ***June*** | **10.22** | **15.44** |  | ***June*** | **1.79** | **13.39** | ***June*** | **10.24** | **15.33** |
|  | ***July*** | **18.27** | **22.28** | ***July*** | **15.40** | **19.35** |  | ***July*** | **5.95** | **14.59** | ***July*** | **15.06** | **18.96** |
|  | ***August*** | **19.48** | **23.11** | ***August*** | **16.62** | **20.29** |  | ***August*** | **6.68** | **14.72** | ***August*** | **15.91** | **19.62** |
|  | ***September*** | **15.47** | **20.03** | ***September*** | **12.62** | **17.28** |  | ***September*** | **3.11** | **13.64** | ***September*** | **11.66** | **16.46** |
|  | ***October*** | 7.45 | 15.11 | ***October*** | 4.60 | 12.81 |  | ***October*** | -3.08 | 13.50 | ***October*** | 4.44 | 12.57 |
|  | ***November*** | -1.22 | 13.18 | ***November*** | -4.12 | 12.68 |  | ***November*** | -9.18 | 15.86 | ***November*** | -2.63 | 12.27 |
|  | ***December*** | -8.38 | 15.17 | ***December*** | -11.34 | 16.39 |  | ***December*** | -15.24 | 19.78 | ***December*** | -9.78 | 15.38 |

|  | **Table S4.** The statistical assessment of multi-ensembled GCM models of T_max_ and T_min_ for each month in Kazakhstan between 1959 and 2021. | | | | | | | | | | | | |
| --- | --- | --- | --- | --- | --- | --- | --- | --- | --- | --- | --- | --- | --- |
| **Tmax** | **Raw multi-ensembled GCM models** | | | **Bias-corrected multi-ensembled GCM models** | | | **Tmin** | **Raw multi-ensembled GCM models** | | | **Bias-corrected multi-ensembled GCM models** | | |
|  | **Month** | **Bias** | **RMSE** | **Month** | **Bias** | **RMSE** |  | **Month** | **Bias** | **RMSE** | **Month** | **Bias** | **RMSE** |
|  | ***January*** | -11.22 | 17.64 | ***January*** | -17.59 | 22.03 |  | ***January*** | -18.68 | 23.16 | ***January*** | -17.58 | 22.03 |
|  | ***February*** | -11.17 | 17.39 | ***February*** | -17.52 | 21.83 |  | ***February*** | -19.58 | 23.65 | ***February*** | -18.54 | 22.55 |
|  | ***March*** | -6.69 | 14.92 | ***March*** | -12.80 | 18.24 |  | ***March*** | -15.36 | 20.23 | ***March*** | -13.47 | 18.58 |
|  | ***April*** | 1.08 | 13.54 | ***April*** | -4.67 | 13.85 |  | ***April*** | -8.05 | 15.47 | ***April*** | -4.86 | 13.76 |
|  | ***May*** | 9.92 | 16.66 | ***May*** | 4.53 | 13.74 |  | ***May*** | -0.37 | 13.15 | ***May*** | 4.16 | 13.44 |
|  | ***June*** | 17.58 | 22.09 | ***June*** | 12.53 | 18.08 |  | ***June*** | 6.26 | 14.69 | ***June*** | 11.98 | 17.59 |
|  | ***July*** | 22.49 | 26.23 | ***July*** | 17.69 | 22.06 |  | ***July*** | 10.65 | 17.08 | ***July*** | 17.19 | 21.52 |
|  | ***August*** | 23.02 | 26.64 | ***August*** | 18.28 | 22.51 |  | ***August*** | 11.10 | 17.36 | ***August*** | 17.73 | 21.96 |
|  | ***September*** | 18.41 | 22.94 | ***September*** | 13.48 | 19.02 |  | ***September*** | 7.12 | 15.42 | ***September*** | 12.96 | 18.52 |
|  | ***October*** | 9.75 | 17.02 | ***October*** | 4.44 | 14.37 |  | ***October*** | 0.29 | 13.90 | ***October*** | 4.88 | 14.37 |
|  | ***November*** | 0.54 | 14.06 | ***November*** | -5.24 | 14.70 |  | ***November*** | -6.55 | 15.51 | ***November*** | -3.21 | 14.19 |
|  | ***December*** | -6.96 | 15.39 | ***December*** | -13.14 | 18.76 |  | ***December*** | -13.30 | 19.18 | ***December*** | -11.23 | 17.56 |

|  | **Table S5.** The statistical assessment of multi-ensembled GCM models of T_max_ and T_min_ for each month in Kyrgyzstan between 1959 and 2021. | | | | | | | | | | | | |
| --- | --- | --- | --- | --- | --- | --- | --- | --- | --- | --- | --- | --- | --- |
| **Tmax** | **Raw multi-ensembled GCM models** | | | **Bias-corrected multi-ensembled GCM models** | | | **Tmin** | **Raw multi-ensembled GCM models** | | | **Bias-corrected multi-ensembled GCM models** | | |
|  | **Month** | **Bias** | **RMSE** | **Month** | **Bias** | **RMSE** |  | **Month** | **Bias** | **RMSE** | **Month** | **Bias** | **RMSE** |
|  | ***January*** | -11.50 | 15.77 | ***January*** | -13.08 | 16.65 |  | ***January*** | -19.28 | 22.06 | ***January*** | -13.63 | 17.00 |
|  | ***February*** | -11.61 | 15.74 | ***February*** | -13.19 | 16.63 |  | ***February*** | -19.96 | 22.57 | ***February*** | -14.33 | 17.48 |
|  | ***March*** | -8.53 | 13.64 | ***March*** | -10.16 | 14.18 |  | ***March*** | -16.76 | 19.79 | ***March*** | -10.30 | 14.28 |
|  | ***April*** | -3.02 | 11.26 | ***April*** | -4.79 | 10.72 |  | ***April*** | -11.32 | 15.37 | ***April*** | -3.81 | 10.27 |
|  | ***May*** | 3.43 | 11.55 | ***May*** | 1.49 | 9.77 |  | ***May*** | -5.72 | 12.13 | ***May*** | 2.83 | 9.88 |
|  | ***June*** | 10.06 | 15.03 | ***June*** | 7.95 | 12.76 |  | ***June*** | -0.84 | 11.35 | ***June*** | 8.56 | 13.02 |
|  | ***July*** | 15.13 | 18.84 | ***July*** | 12.90 | 16.47 |  | ***July*** | 2.70 | 11.91 | ***July*** | 12.68 | 16.19 |
|  | ***August*** | 16.91 | 20.13 | ***August*** | 14.69 | 17.80 |  | ***August*** | 3.55 | 11.94 | ***August*** | 13.70 | 16.93 |
|  | ***September*** | 13.88 | 17.72 | ***September*** | 11.75 | 15.51 |  | ***September*** | 0.61 | 11.42 | ***September*** | 10.12 | 14.20 |
|  | ***October*** | 6.84 | 13.12 | ***October*** | 4.90 | 11.26 |  | ***October*** | -4.53 | 11.95 | ***October*** | 4.06 | 10.71 |
|  | ***November*** | -1.19 | 11.18 | ***November*** | -2.95 | 10.50 |  | ***November*** | -9.54 | 14.39 | ***November*** | -1.75 | 10.15 |
|  | ***December*** | -7.86 | 13.31 | ***December*** | -9.51 | 13.81 |  | ***December*** | -14.83 | 18.26 | ***December*** | -8.15 | 12.89 |

|  | **Table S6.** The statistical assessment of multi-ensembled GCM models of T_max_ and T_min_ for each month in Tajikistan between 1959 and 2021. | | | | | | | | | | | | |
| --- | --- | --- | --- | --- | --- | --- | --- | --- | --- | --- | --- | --- | --- |
| **Tmax** | **Raw multi-ensembled GCM models** | | | **Bias-corrected multi-ensembled GCM models** | | | **Tmin** | **Raw multi-ensembled GCM models** | | | **Bias-corrected multi-ensembled GCM models** | | |
|  | **Month** | **Bias** | **RMSE** | **Month** | **Bias** | **RMSE** |  | **Month** | **Bias** | **RMSE** | **Month** | **Bias** | **RMSE** |
|  | ***January*** | -11.69 | 16.20 | ***January*** | -13.34 | 17.04 |  | ***January*** | -19.36 | 22.29 | ***January*** | -13.38 | 16.94 |
|  | ***February*** | -11.65 | 16.04 | ***February*** | -13.29 | 16.87 |  | ***February*** | -19.75 | 22.56 | ***February*** | -13.81 | 17.19 |
|  | ***March*** | -8.50 | 13.78 | ***March*** | -10.34 | 14.30 |  | ***March*** | -17.04 | 20.07 | ***March*** | -10.41 | 14.30 |
|  | ***April*** | -2.97 | 11.69 | ***April*** | -5.13 | 11.08 |  | ***April*** | -12.00 | 16.15 | ***April*** | -4.38 | 10.69 |
|  | ***May*** | 3.70 | 12.17 | ***May*** | 1.16 | 10.03 |  | ***May*** | -6.17 | 12.85 | ***May*** | 2.60 | 10.10 |
|  | ***June*** | 10.56 | 15.68 | ***June*** | 7.57 | 12.66 |  | ***June*** | -1.24 | 11.88 | ***June*** | 8.50 | 13.11 |
|  | ***July*** | 16.45 | 20.07 | ***July*** | 13.10 | 16.62 |  | ***July*** | 2.61 | 12.38 | ***July*** | 13.06 | 16.46 |
|  | ***August*** | 18.47 | 21.66 | ***August*** | 15.05 | 18.19 |  | ***August*** | 3.66 | 12.65 | ***August*** | 14.32 | 17.51 |
|  | ***September*** | 15.09 | 18.85 | ***September*** | 11.92 | 15.65 |  | ***September*** | 0.56 | 12.08 | ***September*** | 10.51 | 14.50 |
|  | ***October*** | 7.80 | 13.85 | ***October*** | 5.10 | 11.28 |  | ***October*** | -5.07 | 12.65 | ***October*** | 3.81 | 10.55 |
|  | ***November*** | -0.27 | 11.53 | ***November*** | -2.49 | 10.45 |  | ***November*** | -10.26 | 15.13 | ***November*** | -2.31 | 10.25 |
|  | ***December*** | -7.46 | 13.50 | ***December*** | -9.32 | 13.89 |  | ***December*** | -15.37 | 18.89 | ***December*** | -8.50 | 13.25 |

|  | **Table S7.** The statistical assessment of multi-ensembled GCM models of T_max_ and T_min_ for each month in Uzbekistan between 1959 and 2021. | | | | | | | | | | | | |
| --- | --- | --- | --- | --- | --- | --- | --- | --- | --- | --- | --- | --- | --- |
| **Tmax** | **Raw multi-ensembled GCM models** | | | **Bias-corrected multi-ensembled GCM models** | | | **Tmin** | **Raw multi-ensembled GCM models** | | | **Bias-corrected multi-ensembled GCM models** | | |
|  | **Month** | **Bias** | **RMSE** | **Month** | **Bias** | **RMSE** |  | **Month** | **Bias** | **RMSE** | **Month** | **Bias** | **RMSE** |
|  | ***January*** | -13.67 | 17.83 | ***January*** | -13.98 | 17.76 |  | ***January*** | -20.74 | 23.58 | ***January*** | -14.04 | 17.74 |
|  | ***February*** | -13.50 | 17.55 | ***February*** | -13.83 | 17.47 |  | ***February*** | -21.02 | 23.71 | ***February*** | -14.37 | 17.84 |
|  | ***March*** | -10.09 | 15.11 | ***March*** | -10.58 | 14.79 |  | ***March*** | -18.14 | 21.03 | ***March*** | -10.68 | 14.81 |
|  | ***April*** | -4.34 | 12.57 | ***April*** | -5.06 | 11.30 |  | ***April*** | -13.12 | 16.82 | ***April*** | -4.46 | 10.95 |
|  | ***May*** | 2.59 | 12.42 | ***May*** | 1.62 | 10.38 |  | ***May*** | -7.20 | 13.23 | ***May*** | 2.83 | 10.47 |
|  | ***June*** | 9.86 | 15.67 | ***June*** | 8.60 | 13.66 |  | ***June*** | -1.83 | 12.10 | ***June*** | 9.37 | 14.02 |
|  | ***July*** | 15.35 | 19.49 | ***July*** | 13.90 | 17.53 |  | ***July*** | 2.02 | 12.40 | ***July*** | 14.02 | 17.51 |
|  | ***August*** | 16.89 | 20.53 | ***August*** | 15.47 | 18.73 |  | ***August*** | 2.77 | 12.38 | ***August*** | 14.96 | 18.26 |
|  | ***September*** | 13.35 | 17.79 | ***September*** | 12.12 | 16.13 |  | ***September*** | -0.66 | 12.02 | ***September*** | 10.68 | 14.99 |
|  | ***October*** | 5.86 | 13.40 | ***October*** | 4.92 | 11.81 |  | ***October*** | -6.57 | 13.15 | ***October*** | 3.53 | 11.12 |
|  | ***November*** | -2.40 | 12.24 | ***November*** | -3.06 | 11.19 |  | ***November*** | -11.77 | 16.16 | ***November*** | -2.76 | 11.09 |
|  | ***December*** | -9.67 | 15.13 | ***December*** | -10.12 | 14.80 |  | ***December*** | -16.83 | 20.18 | ***December*** | -9.08 | 14.08 |

|  | **Table S8.** The statistical assessment of multi-ensembled GCM models of T_max_ and T_min_ for each month in Turkmenistan between 1959 and 2021. | | | | | | | | | | | | |
| --- | --- | --- | --- | --- | --- | --- | --- | --- | --- | --- | --- | --- | --- |
| **Tmax** | **Raw multi-ensembled GCM models** | | | **Bias-corrected multi-ensembled GCM models** | | | **Tmin** | **Raw multi-ensembled GCM models** | | | **Bias-corrected multi-ensembled GCM models** | | |
|  | **Month** | **Bias** | **RMSE** | **Month** | **Bias** | **RMSE** |  | **Month** | **Bias** | **RMSE** | **Month** | **Bias** | **RMSE** |
|  | ***January*** | -15.17 | 19.62 | ***January*** | -15.00 | 18.88 |  | ***January*** | -22.77 | 25.81 | ***January*** | -14.28 | 18.33 |
|  | ***February*** | -14.98 | 19.17 | ***February*** | -14.81 | 18.42 |  | ***February*** | -23.29 | 25.97 | ***February*** | -14.82 | 18.35 |
|  | ***March*** | -10.80 | 16.09 | ***March*** | -10.69 | 14.94 |  | ***March*** | -20.33 | 23.09 | ***March*** | -11.38 | 15.29 |
|  | ***April*** | -4.63 | 13.83 | ***April*** | -4.67 | 11.72 |  | ***April*** | -14.98 | 18.90 | ***April*** | -5.20 | 11.76 |
|  | ***May*** | 2.97 | 13.93 | ***May*** | 2.67 | 11.35 |  | ***May*** | -8.16 | 14.77 | ***May*** | 2.62 | 11.19 |
|  | ***June*** | 10.25 | 17.05 | ***June*** | 9.74 | 14.75 |  | ***June*** | -1.95 | 13.20 | ***June*** | 9.77 | 14.70 |
|  | ***July*** | 15.82 | 20.35 | ***July*** | 15.19 | 18.70 |  | ***July*** | 2.82 | 13.28 | ***July*** | 15.19 | 18.60 |
|  | ***August*** | 16.85 | 20.99 | ***August*** | 16.26 | 19.73 |  | ***August*** | 3.68 | 13.44 | ***August*** | 16.11 | 19.51 |
|  | ***September*** | 12.25 | 17.81 | ***September*** | 11.78 | 16.22 |  | ***September*** | -0.43 | 12.84 | ***September*** | 11.38 | 15.84 |
|  | ***October*** | 4.39 | 14.63 | ***October*** | 4.14 | 12.18 |  | ***October*** | -7.03 | 14.43 | ***October*** | 3.81 | 11.95 |
|  | ***November*** | -3.78 | 14.64 | ***November*** | -3.82 | 12.60 |  | ***November*** | -13.34 | 18.48 | ***November*** | -3.46 | 12.46 |
|  | ***December*** | -10.88 | 17.25 | ***December*** | -10.79 | 16.18 |  | ***December*** | -18.79 | 22.75 | ***December*** | -9.75 | 15.57 |

| **Table S9.**  The statistics of calculated climate indices separately in studied countries between 1959 and 2100 under both climate scenarios. | | | | | | | | | | | | | | | | | | | |
| --- | --- | --- | --- | --- | --- | --- | --- | --- | --- | --- | --- | --- | --- | --- | --- | --- | --- | --- | --- |
|  | ***Historical Period (1959-2021)*** | | | | | | | | |  | ***Historical Period (1959-2021)*** | | | | | | | | |
|  | **Country** | **Value** | **HWM** | **HWA** | **HWN** | **HWD** | **HWF** | **TX90p** | **TN90p** |  | **Country** | **Value** | **HWM** | **HWA** | **HWN** | **HWD** | **HWF** | **TX90p** | **TN90p** |
|  | **Turkmenistan** | Avg. | 31.9 | 34.7 | NA | NA | NA | 12.9 | 13.0 |  | **Turkmenistan** | Avg. | 31.9 | 34.7 | NA | NA | NA | 12.9 | 13.0 |
|  |  | STD | 2.8 | 3.2 | NA | NA | NA | 5.2 | 5.3 |  |  | STD | 2.8 | 3.2 | NA | NA | NA | 5.2 | 5.3 |
|  |  | Sum | NA | NA | 2658.0 | 5249.0 | 11978.0 | NA | NA |  |  | Sum | NA | NA | 2658.0 | 5249.0 | 11978.0 | NA | NA |
|  |  | Max | 38.3 | 41.2 | NA | NA | NA | 33.2 | 32.3 |  |  | Max | 38.3 | 41.2 | NA | NA | NA | 33.2 | 32.3 |
|  |  | Min | 23.2 | 24.1 | NA | NA | NA | 1.5 | 1.1 |  |  | Min | 23.2 | 24.1 | NA | NA | NA | 1.5 | 1.1 |
|  | ***Near-Future (2022-2051)*** | | | | | | | | |  | ***Near-Future (2022-2051)*** | | | | | | | | |
| ***SSP2-4.5*** | **Turkmenistan** | **Value** | **HWM** | **HWA** | **HWN** | **HWD** | **HWF** | **TX90p** | **TN90p** | ***SSP5–8.5*** | **Turkmenistan** | **Value** | **HWM** | **HWA** | **HWN** | **HWD** | **HWF** | **TX90p** | **TN90p** |
|  |  | Avg. | 26.0 | 28.4 | NA | NA | NA | 13.4 | 14.9 |  |  | Avg. | 29.7 | 33.0 | NA | NA | NA | 13.6 | 18.3 |
|  |  | STD | 2.4 | 2.4 | NA | NA | NA | 7.4 | 11.5 |  |  | STD | 5.8 | 6.3 | NA | NA | NA | 7.2 | 14.4 |
|  |  | Sum | NA | NA | 586.0 | 1018.0 | 2388.0 | NA | NA |  |  | Sum | NA | NA | 1008.0 | 2023.0 | 5013.0 | NA | NA |
|  |  | Max | 29.5 | 31.1 | NA | NA | NA | 32.2 | 50.4 |  |  | Max | 47.0 | 49.8 | NA | NA | NA | 34.9 | 55.3 |
|  |  | Min | 14.9 | 15.2 | NA | NA | NA | 1.6 | 0.9 |  |  | Min | 17.8 | 18.1 | NA | NA | NA | 0.5 | 1.4 |
|  | ***Far-Future (2071-2100)*** | | | | | | | | |  | ***Far-Future (2071-2100)*** | | | | | | | | |
|  | **Turkmenistan** | **Value** | **HWM** | **HWA** | **HWN** | **HWD** | **HWF** | **TX90p** | **TN90p** |  | **Turkmenistan** | **Value** | **HWM** | **HWA** | **HWN** | **HWD** | **HWF** | **TX90p** | **TN90p** |
|  |  | Avg. | 32.0 | 34.3 | NA | NA | NA | 11.0 | 13.3 |  |  | Avg. | 34.7 | 38.3 | NA | NA | NA | 14.2 | 18.1 |
|  |  | STD | 5.7 | 5.7 | NA | NA | NA | 4.5 | 5.1 |  |  | STD | 5.0 | 5.3 | NA | NA | NA | 9.0 | 16.4 |
|  |  | Sum | NA | NA | 852.0 | 1859.0 | 3858.0 | NA | NA |  |  | Sum | NA | NA | 1331.0 | 2875.0 | 7146.0 | NA | NA |
|  |  | Max | 46.4 | 48.0 | NA | NA | NA | 20.8 | 27.9 |  |  | Max | 50.5 | 53.7 | NA | NA | NA | 34.5 | 55.6 |
|  |  | Min | 18.7 | 19.4 | NA | NA | NA | 0.8 | 4.6 |  |  | Min | 25.7 | 26.8 | NA | NA | NA | 0.8 | 0.0 |
|  | ***Historical Period (1959-2021)*** | | | | | | | | |  | ***Historical Period (1959-2021)*** | | | | | | | | |
|  | **Tajikistan** | **Value** | **HWM** | **HWA** | **HWN** | **HWD** | **HWF** | **TX90p** | **TN90p** |  | **Tajikistan** | **Value** | **HWM** | **HWA** | **HWN** | **HWD** | **HWF** | **TX90p** | **TN90p** |
|  |  | Avg. | 24.7 | 27.6 | NA | NA | NA | 13.1 | 13.1 |  |  | Avg. | 24.7 | 27.6 | NA | NA | NA | 13.1 | 13.1 |
|  |  | STD | 6.4 | 6.6 | NA | NA | NA | 6.3 | 6.3 |  |  | STD | 6.4 | 6.6 | NA | NA | NA | 6.3 | 6.3 |
|  |  | Sum | NA | NA | 13409.0 | 29685.0 | 66703.0 | NA | NA |  |  | Sum | NA | NA | 13409.0 | 29685.0 | 66703.0 | NA | NA |
|  |  | Max | 37.6 | 39.7 | NA | NA | NA | 44.4 | 42.7 |  |  | Max | 37.6 | 39.7 | NA | NA | NA | 44.4 | 42.7 |
|  |  | Min | -2.2 | -1.6 | NA | NA | NA | 0.3 | 0.3 |  |  | Min | -2.2 | -1.6 | NA | NA | NA | 0.3 | 0.3 |
|  | ***Near-Future (2022-2051)*** | | | | | | | | |  | ***Near-Future (2022-2051)*** | | | | | | | | |
| ***SSP2-4.5*** | **Tajikistan** | **Value** | **HWM** | **HWA** | **HWN** | **HWD** | **HWF** | **TX90p** | **TN90p** | ***SSP5–8.5*** | **Tajikistan** | **Value** | **HWM** | **HWA** | **HWN** | **HWD** | **HWF** | **TX90p** | **TN90p** |
|  |  | Avg. | 26.3 | 28.6 | NA | NA | NA | 13.4 | 15.0 |  |  | Avg. | 28.1 | 31.5 | NA | NA | NA | 13.6 | 18.5 |
|  |  | STD | 2.3 | 2.1 | NA | NA | NA | 7.4 | 11.7 |  |  | STD | 4.3 | 4.9 | NA | NA | NA | 7.4 | 14.5 |
|  |  | Sum | NA | NA | 3894.0 | 6534.0 | 15790.0 | NA | NA |  |  | Sum | NA | NA | 6059.0 | 11997.0 | 30325.0 | NA | NA |
|  |  | Max | 29.6 | 31.2 | NA | NA | NA | 32.8 | 51.8 |  |  | Max | 39.4 | 42.3 | NA | NA | NA | 35.2 | 55.9 |
|  |  | Min | 15.2 | 15.5 | NA | NA | NA | 1.6 | 0.5 |  |  | Min | 17.9 | 18.0 | NA | NA | NA | 0.4 | 1.1 |
|  | ***Far-Future (2071-2100)*** | | | | | | | | |  | ***Far-Future (2071-2100)*** | | | | | | | | |
|  | **Tajikistan** | **Value** | **HWM** | **HWA** | **HWN** | **HWD** | **HWF** | **TX90p** | **TN90p** |  | **Tajikistan** | **Value** | **HWM** | **HWA** | **HWN** | **HWD** | **HWF** | **TX90p** | **TN90p** |
|  |  | Avg. | 30.2 | 32.3 | NA | NA | NA | 10.9 | 13.3 |  |  | Avg. | 33.1 | 36.7 | NA | NA | NA | 14.3 | 18.4 |
|  |  | STD | 4.6 | 4.5 | NA | NA | NA | 4.5 | 5.2 |  |  | STD | 3.8 | 3.8 | NA | NA | NA | 9.3 | 16.9 |
|  |  | Sum | NA | NA | 4484.0 | 10359.0 | 20721.0 | NA | NA |  |  | Sum | NA | NA | 6916.0 | 14724.0 | 37669.0 | NA | NA |
|  |  | Max | 41.0 | 41.9 | NA | NA | NA | 21.4 | 27.6 |  |  | Max | 44.3 | 46.0 | NA | NA | NA | 34.5 | 55.9 |
|  |  | Min | 18.3 | 19.0 | NA | NA | NA | 0.5 | 4.4 |  |  | Min | 23.9 | 24.4 | NA | NA | NA | 0.8 | 0.0 |
|  | ***Historical Period (1959-2021)*** | | | | | | | | |  | ***Historical Period (1959-2021)*** | | | | | | | | |
|  | **Kazakhstan** | **Value** | **HWM** | **HWA** | **HWN** | **HWD** | **HWF** | **TX90p** | **TN90p** |  | **Kazakhstan** | **Value** | **HWM** | **HWA** | **HWN** | **HWD** | **HWF** | **TX90p** | **TN90p** |
|  |  | Avg. | 26.1 | 29.3 | NA | NA | NA | 12.6 | 12.6 |  |  | Avg. | 26.1 | 29.3 | NA | NA | NA | 12.6 | 12.6 |
|  |  | STD | 3.9 | 4.3 | NA | NA | NA | 5.7 | 5.7 |  |  | STD | 3.9 | 4.3 | NA | NA | NA | 5.7 | 5.7 |
|  |  | Sum | NA | NA | 44178.0 | 92725.0 | 206532.0 | NA | NA |  |  | Sum | NA | NA | 44178.0 | 92725.0 | 206532.0 | NA | NA |
|  |  | Max | 36.3 | 40.4 | NA | NA | NA | 34.8 | 36.2 |  |  | Max | 36.3 | 40.4 | NA | NA | NA | 34.8 | 36.2 |
|  |  | Min | 10.5 | 11.0 | NA | NA | NA | 0.3 | 0.3 |  |  | Min | 10.5 | 11.0 | NA | NA | NA | 0.3 | 0.3 |
|  | ***Near-Future (2022-2051)*** | | | | | | | | |  | ***Near-Future (2022-2051)*** | | | | | | | | |
| ***SSP2-4.5*** | **Kazakhstan** | **Value** | **HWM** | **HWA** | **HWN** | **HWD** | **HWF** | **TX90p** | **TN90p** | ***SSP5–8.5*** | **Kazakhstan** | **Value** | **HWM** | **HWA** | **HWN** | **HWD** | **HWF** | **TX90p** | **TN90p** |
|  |  | Avg. | 26.2 | 28.6 | NA | NA | NA | 13.4 | 14.9 |  |  | Avg. | 26.5 | 30.0 | NA | NA | NA | 13.6 | 18.4 |
|  |  | STD | 2.1 | 1.9 | NA | NA | NA | 7.4 | 11.7 |  |  | STD | 3.5 | 4.5 | NA | NA | NA | 7.4 | 14.4 |
|  |  | Sum | NA | NA | 18328.0 | 31306.0 | 74564.0 | NA | NA |  |  | Sum | NA | NA | 21819.0 | 43248.0 | 108290.0 | NA | NA |
|  |  | Max | 38.5 | 40.8 | NA | NA | NA | 34.4 | 54.0 |  |  | Max | 40.6 | 43.3 | NA | NA | NA | 35.2 | 57.3 |
|  |  | Min | 14.5 | 14.8 | NA | NA | NA | 1.6 | 0.5 |  |  | Min | 16.9 | 17.3 | NA | NA | NA | 0.2 | 1.6 |
|  | ***Far-Future (2071-2100)*** | | | | | | | | |  | ***Far-Future (2071-2100)*** | | | | | | | | |
|  | **Kazakhstan** | **Value** | **HWM** | **HWA** | **HWN** | **HWD** | **HWF** | **TX90p** | **TN90p** |  | **Kazakhstan** | **Value** | **HWM** | **HWA** | **HWN** | **HWD** | **HWF** | **TX90p** | **TN90p** |
|  |  | Avg. | 28.5 | 30.8 | NA | NA | NA | 11.0 | 13.5 |  |  | Avg. | 31.9 | 35.5 | NA | NA | NA | 14.3 | 18.4 |
|  |  | STD | 3.4 | 3.4 | NA | NA | NA | 4.6 | 5.4 |  |  | STD | 3.1 | 3.2 | NA | NA | NA | 9.3 | 16.9 |
|  |  | Sum | NA | NA | 16612.0 | 39100.0 | 77430.0 | NA | NA |  |  | Sum | NA | NA | 26824.0 | 55797.0 | 143662.0 | NA | NA |
|  |  | Max | 41.0 | 43.2 | NA | NA | NA | 22.1 | 27.9 |  |  | Max | 46.2 | 53.2 | NA | NA | NA | 34.8 | 59.3 |
|  |  | Min | 17.6 | 17.9 | NA | NA | NA | 0.0 | 4.1 |  |  | Min | 19.1 | 19.5 | NA | NA | NA | 0.8 | 0.0 |
|  | ***Historical Period (1959-2021)*** | | | | | | | | |  | ***Historical Period (1959-2021)*** | | | | | | | | |
|  | **Kyrgyzstan** | **Value** | **HWM** | **HWA** | **HWN** | **HWD** | **HWF** | **TX90p** | **TN90p** |  | **Kyrgyzstan** | **Value** | **HWM** | **HWA** | **HWN** | **HWD** | **HWF** | **TX90p** | **TN90p** |
|  |  | Avg. | 21.6 | 24.3 | NA | NA | NA | 12.7 | 12.7 |  |  | Avg. | 21.63 | 24.30 | NA | NA | NA | 12.67 | 12.69 |
|  |  | STD | 4.9 | 5.3 | NA | NA | NA | 5.0 | 5.0 |  |  | STD | 4.95 | 5.33 | NA | NA | NA | 4.95 | 5.02 |
|  |  | Sum | NA | NA | 35316.0 | 73271.0 | 158157.0 | NA | NA |  |  | Sum | NA | NA | 35316.00 | 73271.00 | 158157.00 | NA | NA |
|  |  | Max | 34.2 | 37.4 | NA | NA | NA | 32.9 | 32.9 |  |  | Max | 34.23 | 37.38 | NA | NA | NA | 32.88 | 32.88 |
|  |  | Min | 4.0 | 4.4 | NA | NA | NA | 1.9 | 1.6 |  |  | Min | 4.03 | 4.36 | NA | NA | NA | 1.88 | 1.62 |
|  | ***Near-Future (2022-2051)*** | | | | | | | | |  | ***Near-Future (2022-2051)*** | | | | | | | | |
| ***SSP2-4.5*** | **Kyrgyzstan** | **Value** | **HWM** | **HWA** | **HWN** | **HWD** | **HWF** | **TX90p** | **TN90p** | ***SSP5–8.5*** | **Tajikistan** | **Value** | **HWM** | **HWA** | **HWN** | **HWD** | **HWF** | **TX90p** | **TN90p** |
|  |  | Avg. | 26.0 | 28.4 | NA | NA | NA | 13.4 | 14.9 |  |  | Avg. | 27.2 | 30.7 | NA | NA | NA | 13.6 | 18.4 |
|  |  | STD | 2.3 | 2.3 | NA | NA | NA | 7.4 | 11.5 |  |  | STD | 4.0 | 4.8 | NA | NA | NA | 7.3 | 14.4 |
|  |  | Sum | NA | NA | 11590.0 | 19633.0 | 47052.0 | NA | NA |  |  | Sum | NA | NA | 16075.0 | 31795.0 | 80343.0 | NA | NA |
|  |  | Max | 31.2 | 32.6 | NA | NA | NA | 33.9 | 51.8 |  |  | Max | 40.2 | 43.4 | NA | NA | NA | 35.2 | 55.9 |
|  |  | Min | 14.5 | 14.8 | NA | NA | NA | 1.6 | 0.7 |  |  | Min | 16.0 | 16.0 | NA | NA | NA | 0.4 | 1.1 |
|  | ***Far-Future (2071-2100)*** | | | | | | | | |  | ***Far-Future (2071-2100)*** | | | | | | | | |
|  | **Kyrgyzstan** | **Value** | **HWM** | **HWA** | **HWN** | **HWD** | **HWF** | **TX90p** | **TN90p** |  | **Kyrgyzstan** | **Value** | **HWM** | **HWA** | **HWN** | **HWD** | **HWF** | **TX90p** | **TN90p** |
|  |  | Avg. | 29.2 | 31.5 | NA | NA | NA | 11.0 | 13.4 |  |  | Avg. | 32.3 | 35.9 | NA | NA | NA | 14.4 | 18.4 |
|  |  | STD | 4.0 | 3.9 | NA | NA | NA | 4.5 | 5.3 |  |  | STD | 3.4 | 3.5 | NA | NA | NA | 9.3 | 16.9 |
|  |  | Sum | NA | NA | 12467.0 | 28511.0 | 57664.0 | NA | NA |  |  | Sum | NA | NA | 19523.0 | 41012.0 | 105059.0 | NA | NA |
|  |  | Max | 40.7 | 41.8 | NA | NA | NA | 22.1 | 27.9 |  |  | Max | 45.3 | 46.4 | NA | NA | NA | 35.3 | 56.4 |
|  |  | Min | 18.1 | 18.7 | NA | NA | NA | 0.3 | 4.1 |  |  | Min | 19.0 | 19.5 | NA | NA | NA | 0.8 | 0.0 |
|  | ***Historical Period (1959-2021)*** | | | | | | | | |  | ***Historical Period (1959-2021)*** | | | | | | | | |
|  | **Uzbekistan** | **Value** | **HWM** | **HWA** | **HWN** | **HWD** | **HWF** | **TX90p** | **TN90p** |  | **Uzbekistan** | **HWM** | **HWA** | **HWN** | **HWD** | **HWF** | **TX90p** | **TN90p** | **HWM** |
|  |  | Avg. | 28.4 | 31.3 | NA | NA | NA | 12.8 | 12.8 |  |  | Avg. | 28.4 | 31.3 | NA | NA | NA | 12.8 | 12.8 |
|  |  | STD | 3.5 | 4.0 | NA | NA | NA | 5.3 | 5.3 |  |  | STD | 3.5 | 4.0 | NA | NA | NA | 5.3 | 5.3 |
|  |  | Sum | NA | NA | 5574.0 | 12082.0 | 26189.0 | NA | NA |  |  | Sum | NA | NA | 5574.0 | 12082.0 | 26189.0 | NA | NA |
|  |  | Max | 37.5 | 39.6 | NA | NA | NA | 38.8 | 37.7 |  |  | Max | 37.5 | 39.6 | NA | NA | NA | 38.8 | 37.7 |
|  |  | Min | 15.1 | 15.5 | NA | NA | NA | 2.0 | 1.8 |  |  | Min | 15.1 | 15.5 | NA | NA | NA | 2.0 | 1.8 |
|  | ***Near-Future (2022-2051)*** | | | | | | | | |  | ***Near-Future (2022-2051)*** | | | | | | | | |
| ***SSP2-4.5*** | **Uzbekistan** | **Value** | **HWM** | **HWA** | **HWN** | **HWD** | **HWF** | **TX90p** | **TN90p** | ***SSP5–8.5*** | **Tajikistan** | **Value** | **HWM** | **HWA** | **HWN** | **HWD** | **HWF** | **TX90p** | **TN90p** |
|  |  | Avg. | 26.2 | 28.6 | NA | NA | NA | 13.4 | 15.0 |  |  | Avg. | 28.1 | 31.5 | NA | NA | NA | 13.7 | 18.5 |
|  |  | STD | 2.4 | 2.2 | NA | NA | NA | 7.4 | 11.7 |  |  | STD | 4.5 | 5.1 | NA | NA | NA | 7.4 | 14.5 |
|  |  | Sum | NA | NA | 1845.0 | 3160.0 | 7513.0 | NA | NA |  |  | Sum | NA | NA | 2538.0 | 4982.0 | 12681.0 | NA | NA |
|  |  | Max | 29.5 | 31.1 | NA | NA | NA | 33.3 | 51.5 |  |  | Max | 39.6 | 42.1 | NA | NA | NA | 34.9 | 55.9 |
|  |  | Min | 14.8 | 15.1 | NA | NA | NA | 1.6 | 0.7 |  |  | Min | 17.1 | 17.5 | NA | NA | NA | 0.4 | 2.2 |
|  | ***Far-Future (2071-2100)*** | | | | | | | | |  | ***Far-Future (2071-2100)*** | | | | | | | | |
|  | **Uzbekistan** | **Value** | **HWM** | **HWA** | **HWN** | **HWD** | **HWF** | **TX90p** | **TN90p** |  | **Uzbekistan** | **Value** | **HWM** | **HWA** | **HWN** | **HWD** | **HWF** | **TX90p** | **TN90p** |
|  |  | Avg. | 29.8 | 32.1 | NA | NA | NA | 11.0 | 13.4 |  |  | Avg. | 32.9 | 36.5 | NA | NA | NA | 14.4 | 18.4 |
|  |  | STD | 4.7 | 4.6 | NA | NA | NA | 4.5 | 5.3 |  |  | STD | 3.7 | 3.8 | NA | NA | NA | 9.3 | 16.8 |
|  |  | Sum | NA | NA | 2140.0 | 4922.0 | 9860.0 | NA | NA |  |  | Sum | NA | NA | 3339.0 | 7076.0 | 18113.0 | NA | NA |
|  |  | Max | 41.0 | 42.1 | NA | NA | NA | 20.8 | 27.9 |  |  | Max | 44.1 | 46.2 | NA | NA | NA | 34.5 | 55.3 |
|  |  | Min | 18.2 | 18.9 | NA | NA | NA | 0.8 | 4.6 |  |  | Min | 23.9 | 24.5 | NA | NA | NA | 0.8 | 0.0 |

| **Table S10.** The trend analysis for all-cause mortalities associated with heatwaves across CA from 1959 to 2100. | | | | | | | |
| --- | --- | --- | --- | --- | --- | --- | --- |
| **Historical (1959-2021)** | | | | | | | |
|  | **Country** | **p-value** | **Slope** |  | **Country** | **p-value** | **Slope** |
|  | Kazakhstan | **< 0.0001** | **1.267** |  | Kazakhstan | **< 0.0001** | **1.267** |
|  | Kyrgyzstan | **0.002** | 0.172 |  | Kyrgyzstan | **0.002** | 0.172 |
|  | Tajikistan | **< 0.0001** | 0.213 |  | Tajikistan | **< 0.0001** | 0.213 |
|  | Turkmenistan | **< 0.0001** | 0.379 |  | Turkmenistan | **< 0.0001** | 0.379 |
|  | Uzbekistan | **< 0.0001** | 1.078 |  | Uzbekistan | **< 0.0001** | 1.078 |
|  | Central Asia | **< 0.0001** | 3.109 |  | Central Asia | **< 0.0001** | 3.109 |
| **Near-Future (2022-2051)** | | | | | | | |
| ***SSP2–4.5*** | **Country** | **p-value** | **Slope** | ***SSP5–8.5*** | **Country** | **p-value** | **Slope** |
|  | Kazakhstan | **< 0.0001** | **5.432** |  | Kazakhstan | **< 0.0001** | **7.310** |
|  | Kyrgyzstan | **< 0.0001** | 1.414 |  | Kyrgyzstan | **< 0.0001** | 2.077 |
|  | Tajikistan | **< 0.0001** | 1.491 |  | Tajikistan | **< 0.0001** | 2.230 |
|  | Turkmenistan | **< 0.0001** | 1.479 |  | Turkmenistan | **< 0.0001** | 1.709 |
|  | Uzbekistan | **< 0.0001** | 3.021 |  | Uzbekistan | **< 0.0001** | 6.300 |
|  | Central Asia | **< 0.0001** | 12.838 |  | Central Asia | **< 0.0001** | 19.625 |
| **Far-Future (2071-2100)** | | | | | | | |
| ***SSP2–4.5*** | **Country** | **p-value** | **Slope** | ***SSP5–8.5*** | **Country** | **p-value** | **Slope** |
|  | Kazakhstan | 0.363 | **1.377** |  | Kazakhstan | **< 0.0001** | **12.461** |
|  | Kyrgyzstan | 0.270 | 0.631 |  | Kyrgyzstan | **< 0.0001** | 5.651 |
|  | Tajikistan | 0.267 | 0.816 |  | Tajikistan | **< 0.0001** | 4.207 |
|  | Turkmenistan | 0.357 | 0.444 |  | Turkmenistan | **< 0.0001** | 3.420 |
|  | Uzbekistan | 0.457 | 1.102 |  | Uzbekistan | **< 0.0001** | 11.269 |
|  | Central Asia | 0.356 | 4.370 |  | Central Asia | **< 0.0001** | 37.008 |

| **Table S11.**  The total number of heatwaves (HWN) across CA from 1959 to 2100. | | | |
| --- | --- | --- | --- |
| **Historical (1959-2021)** | | | |
| ***Country*** | **HWN** | ***Country*** | **HWN** |
| Kazakhstan | **40162** | Kazakhstan | **40162** |
| Kyrgyzstan | 33636 | Kyrgyzstan | 33636 |
| Tajikistan | 12031 | Tajikistan | 12031 |
| Turkmenistan | 2473 | Turkmenistan | 2473 |
| Uzbekistan | 5425 | Uzbekistan | 5425 |
| Central Asia | 93727 | Central Asia | 93727 |
| ***SSP2–4.5*** | | | |
| **Near-Future (2022-2051)** | | **Far-Future (2071-2100)** | |
| ***Country*** | **HWN** | ***Country*** | **HWN** |
| Kazakhstan | **15702** | Kazakhstan | **14248** |
| Kyrgyzstan | 12338 | Kyrgyzstan | 11839 |
| Tajikistan | 4317 | Tajikistan | 4110 |
| Turkmenistan | 832 | Turkmenistan | 794 |
| Uzbekistan | 1930 | Uzbekistan | 1862 |
| Central Asia | 35119 | Central Asia | 32853 |
| ***SSP5–8.5*** | | | |
| **Near-Future (2022-2051)** | | **Far-Future (2071-2100)** | |
| ***Country*** | **HWN** | ***Country*** | **HWN** |
| Kazakhstan | **18670** | Kazakhstan | **22886** |
| Kyrgyzstan | 15353 | Kyrgyzstan | 18326 |
| Tajikistan | 5423 | Tajikistan | 6410 |
| Turkmenistan | 937 | Turkmenistan | 1253 |
| Uzbekistan | 2164 | Uzbekistan | 2839 |
| Central Asia | 42547 | Central Asia | 51714 |


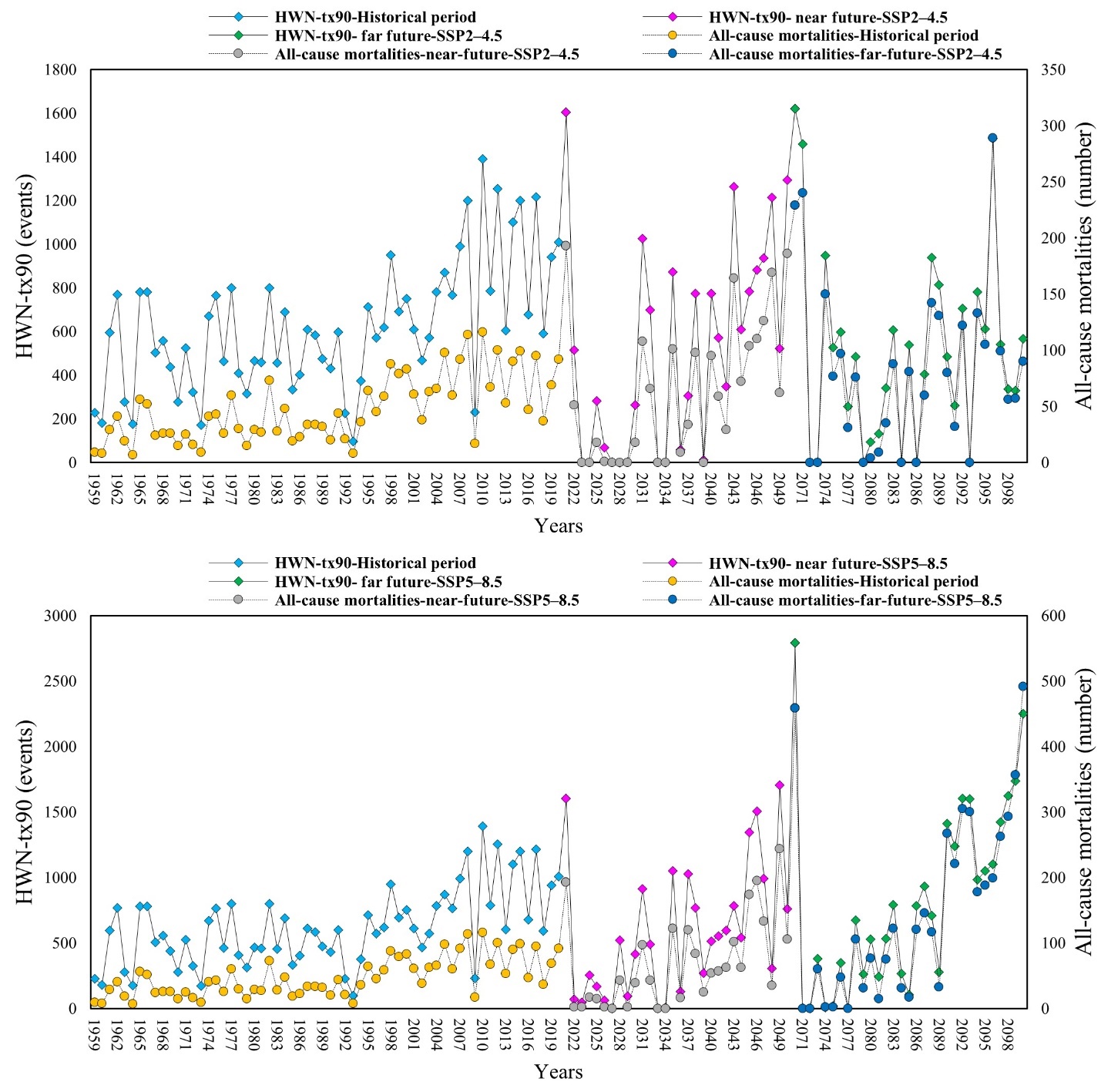
**Fig. S1.** The temporal distribution of HWN-Tx90 and all-cause mortalities between 1959 and 2100 under climate scenarios’ of (**A**) SSP2–4.5, and (**B**) SSP5–8.5 in Kazakhstan.

**B**

**A**


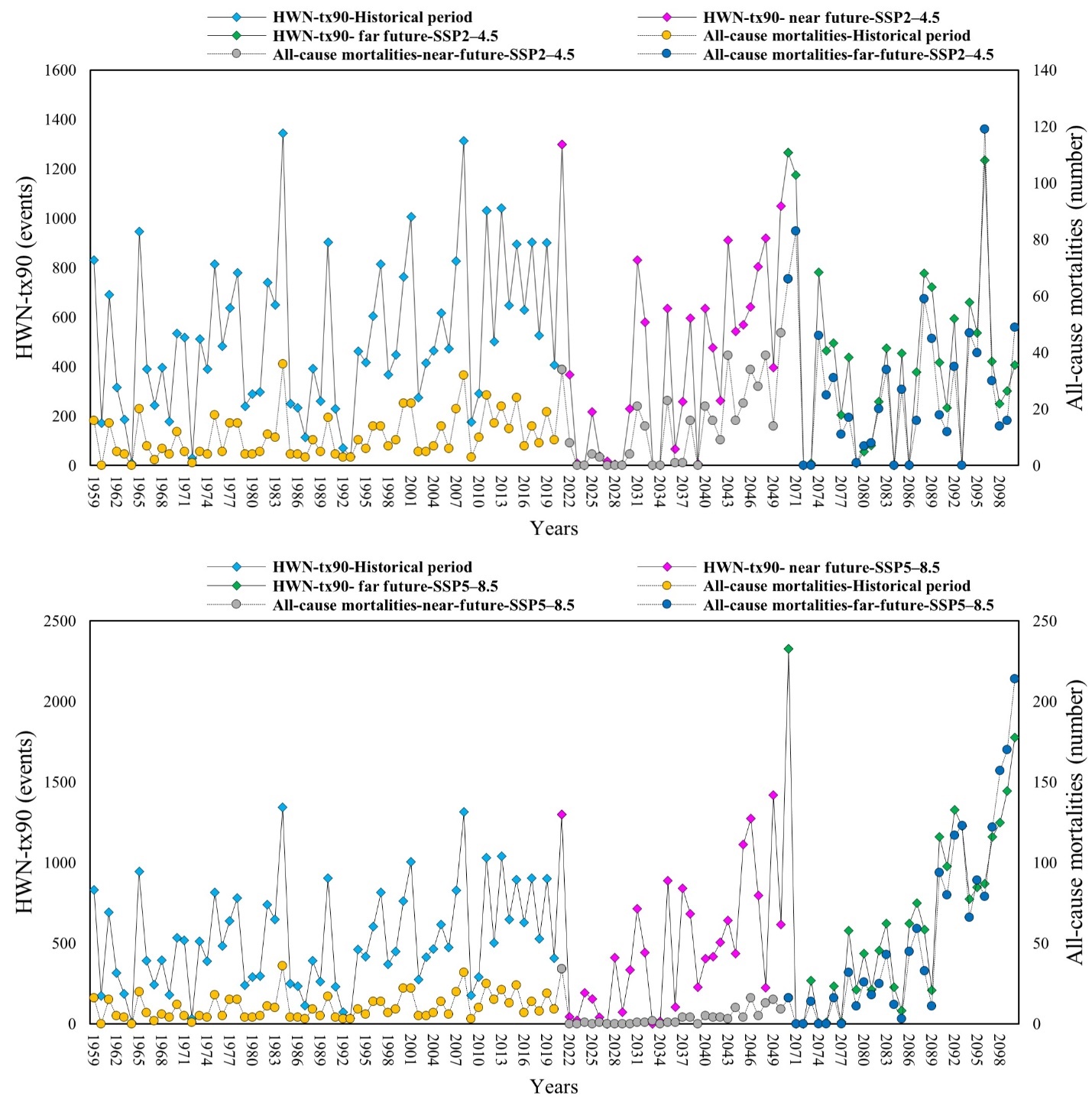
**Fig. S2.** The temporal distribution of HWN-Tx90 and all-cause mortalities between 1959 and 2100 under climate scenarios’ of (**A**) SSP2–4.5, and (**B**) SSP5–8.5 in Kyrgyzstan.

**B**

**A**


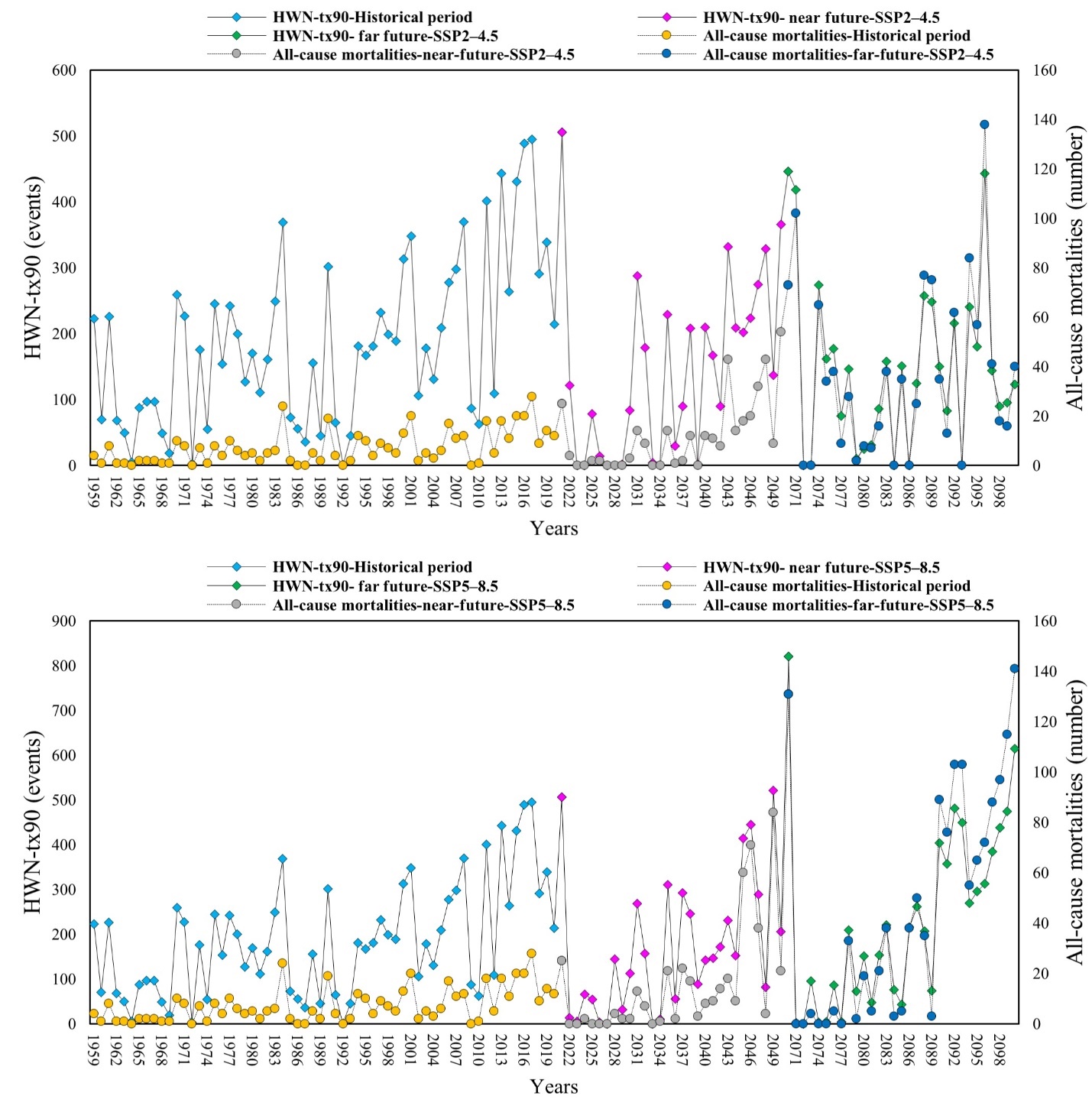


**B**

**A**

**Fig. S3.** The temporal distribution of HWN-Tx90 and all-cause mortalities between 1959 and 2100 under climate scenarios’ of (**A**) SSP2–4.5, and (**B**) SSP5–8.5 in Tajikistan.


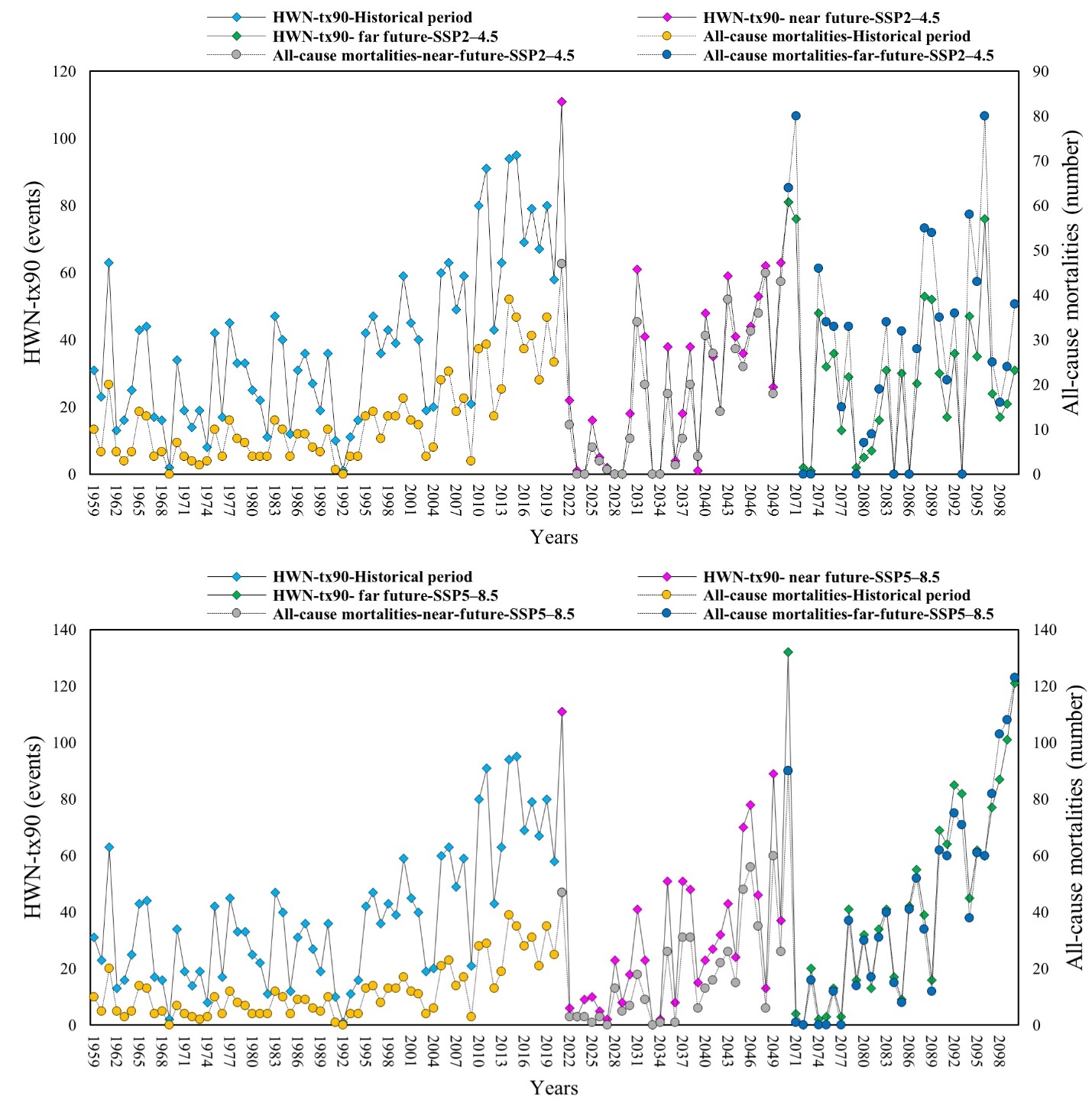
**Fig. S4.** The temporal distribution of HWN-Tx90 and all-cause mortalities between 1959 and 2100 under climate scenarios’ of (**A**) SSP2–4.5, and (**B**) SSP5–8.5 in Turkmenistan.

**B**

**A**


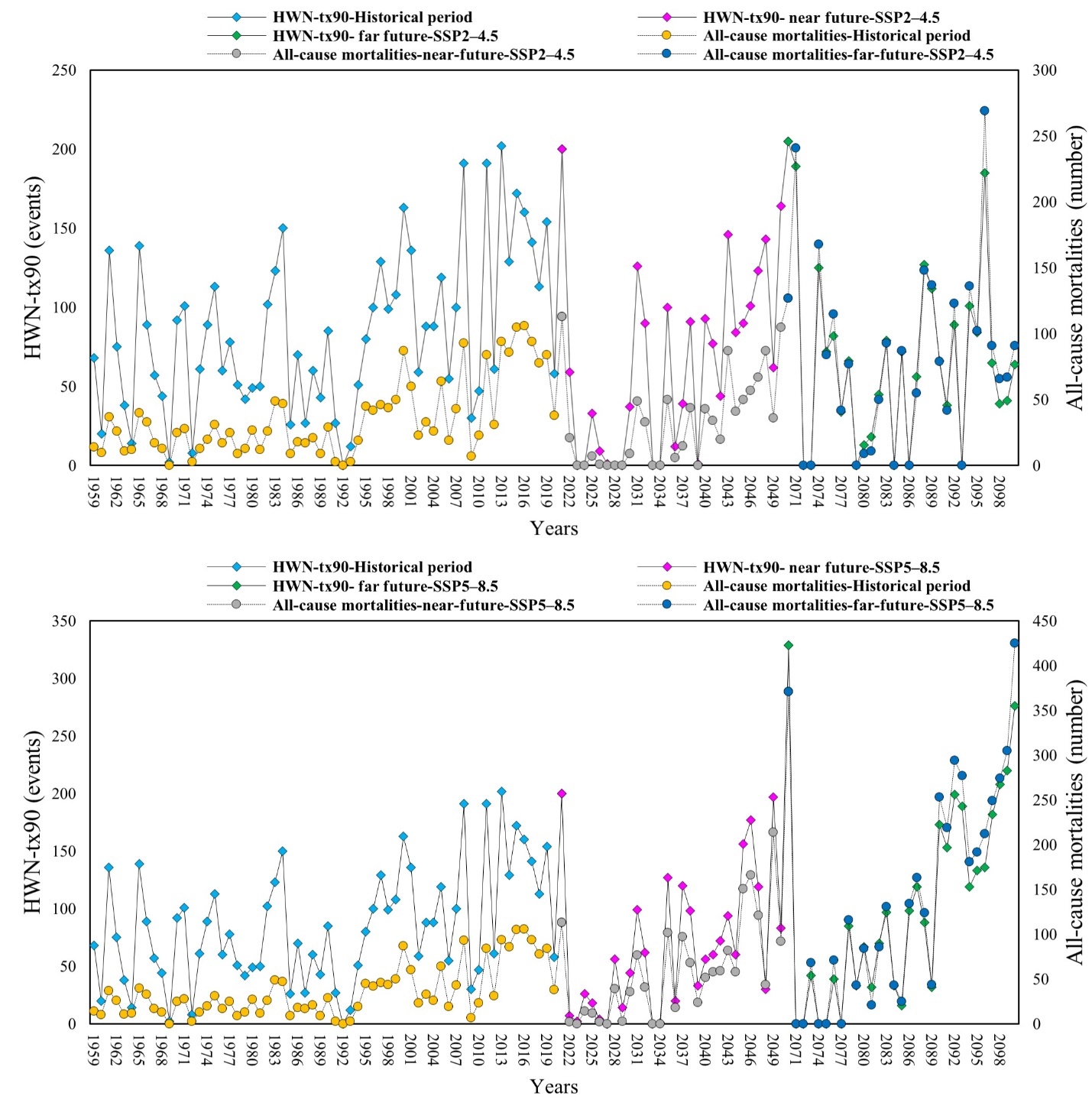


**B**

**A**

**Fig. S5.** The temporal distribution of HWN-Tx90 and all-cause mortalities between 1959 and 2100 under climate scenarios’ of (**A**) SSP2–4.5, and (**B**) SSP5–8.5 in Uzbekistan.
